# Effect of Silver Diamine Fluoride on Caries Arrest and Prevention: The CariedAway School-Based Randomized Clinical Trial

**DOI:** 10.1001/jamanetworkopen.2022.55458

**Published:** 2023-02-09

**Authors:** Ryan Richard Ruff, Tamarinda Barry-Godín, Richard Niederman

**Affiliations:** 1 Department of Epidemiology and Health Promotion, New York University College of Dentistry, New York, New York

## Abstract

**Importance:**

Dental caries is the most common global childhood disease. To control caries, the Centers for Disease Control and Prevention recommends school-based caries prevention, and the World Health Organization lists glass ionomer cement and silver diamine fluoride as essential medicines for oral disease.

**Objective:**

To determine the noninferiority of silver diamine fluoride with fluoride varnish vs traditional glass ionomer sealants with fluoride varnish after 2 years when provided to children via a school-based health care program.

**Design, Setting, and Participants:**

The CariedAway study is an ongoing single-blind, cluster randomized, noninferiority trial conducted between February 1, 2019, and June 1, 2023, among 2998 children in 47 New York City primary schools. Children aged 5 to 13 years of any race and ethnicity were recruited from block-randomized schools. Inclusion criteria for schools were a student population of at least 50% Hispanic or Latino or Latina ethnicity and/or Black race and at least 80% of students receiving free or reduced-cost lunch. Statistical analysis is reported through March 2022.

**Interventions:**

Children received a single application of silver diamine fluoride with fluoride varnish or an active comparator of glass ionomer sealants and atraumatic restorations with fluoride varnish.

**Main Outcomes and Measures:**

Primary outcomes were caries arrest and incidence after a 2-year follow-up, assessed using mixed-effects multilevel models and clustered 2-sample proportion tests. The noninferiority margin was 10%. Intention-to-treat analysis was performed using multiple imputation.

**Results:**

A total of 2998 children (1566 girls [52.2%]; mean [SD] age at baseline, 6.6 [1.2] years; 1397 Hispanic or Latino or Latina children [46.6%]; 874 [29.2%] with untreated dental caries) were recruited and treated from September 16, 2019, to March 12, 2020. Follow-up observations were completed for 1398 children from June 7, 2021, to March 2, 2022. The mean (SE) proportion of children with arrested caries was 0.56 (0.04) after experimental treatment and 0.46 (0.04) after control treatment (difference, −0.11; 95% CI, −0.22 to 0.01). The mean (SE) proportion of patients without new caries was 0.81 (0.02) after experimental treatment and 0.82 (0.02) after control treatment (difference, 0.01; 95% CI, −0.04 to 0.06). Analysis of imputed data for the full sample did not deviate from per-protocol analyses. There were no adverse events.

**Conclusions and Relevance:**

In this randomized clinical trial, silver diamine fluoride with fluoride varnish was noninferior to sealants and atraumatic restorations with fluoride varnish for caries arrest and prevention. Results may support the use of silver diamine fluoride as an arresting and preventive agent in school-based oral health programs.

**Trial Registration:**

ClinicalTrials.gov Identifier: NCT03442309

## Key Points

**Question:**

Is silver diamine fluoride noninferior to dental sealants, the standard of care, for the arrest and prevention of caries in a school-based program?

**Findings:**

In this cluster randomized noninferiority trial, the proportion of children with arrested caries after 2 years was 0.56 among those receiving silver diamine fluoride and 0.46 among those receiving glass ionomer sealants. The proportion of children who remained free from caries was 0.81 among those receiving silver diamine fluoride and 0.82 among those receiving sealants.

**Meaning:**

This study found that silver diamine fluoride was noninferior to traditional sealants when used in a school-based program.

## Teaser

This randomized clinical trial assessed the noninferiority of silver diamine fluoride with fluoride varnish vs traditional glass ionomer sealants with fluoride varnish after 2 years when provided to children via a school-based health care program.

## Introduction

Dental caries (tooth decay) is a natural process by which bacteria in the biofilm cause fluctuations in pH, leading to enamel erosion and a resulting visible lesion.^1^ If left untreated, caries can result in pain, abscess, and systemic infection, leading to functional and/or psychosocial impairment.^2^ Caries is the most prevalent childhood disease in the world and is most prominent among low-income populations.^3^ The disproportionate burden of caries in vulnerable groups stems largely from lower use of dental services; those most at risk often lack access to preventive services or affordable dental care.^4,5^

To reduce children’s caries burden, the Centers for Disease Control and Prevention recommends dental sealants and topical fluorides as part of a school-based caries prevention program.^6,7^ Similarly, the World Health Organization lists silver diamine fluoride (SDF) and glass ionomer cement as essential medicines for dental caries.^8^ The efficacy of these treatments is well established: clinical guidelines for topical fluoride conclude that a 2.3% concentration of fluoride varnish or 1.2% fluoride gel is recommend for children, adolescents, and adults^9^; fluoride varnish had a 70% reduction in demineralized white lesions compared with placebo^10^; dental sealants significantly reduce caries incidence and arrest the progression of noncavitated lesions, showing an 11% reduction in the proportion of carious surfaces when comparing sealants with no sealant^11^; atraumatic restorative treatment noninvasively arrests caries, with median survival times equivalent to those of more traditional restorative intervention^12,13,14^; and SDF reduces the risk of carious lesions and controls caries progression, including a relative risk of 0.6 in the arrest of carious lesions of root surfaces compared with fluoride varnish.^15,16^

Use of alternative medicaments in school-based caries prevention may obviate the financial and workforce barriers known to limit school sealant programs.^17^ For example, SDF is cost effective,^18^ can be applied in less time than dental sealants,^19^ and can be provided by registered nurses. As a result, evidence that SDF is comparable in the treatment of dental caries in a school setting may substantially improve the reach and effectiveness of caries prevention as a dental public health intervention. We conducted the CariedAway school-based pragmatic (conducted in everyday settings)^20^ randomized clinical trial to test the noninferiority of SDF plus fluoride varnish compared with traditional dental sealants and fluoride varnish. We report on the 2-year differences in caries arrest and caries incidence.

## Methods

This study received ethical approval from the New York University School of Medicine institutional review board and is reported following the Consolidated Standards of Reporting Trials (CONSORT) reporting guideline for randomized clinical trials. Parents provided written informed consent, and participants gave oral assent. Detailed study information has been previously published in an available trial protocol^21^ and is included in Supplement 1.

A stakeholder and community advisory board was created to inform the design and conduct of the trial and assist in the interpretation and dissemination of findings. The board consisted of 35 local health and education leaders, including representatives from the New York City Department of Health, researchers, clinicians, school principals, school nurses, teachers, and parents.

### Design and Participants

CariedAway is an ongoing cluster randomized, single-blind, pragmatic noninferiority clinical trial conducted in New York City primary schools between February 1, 2019, and June 1, 2023, to evaluate the effectiveness of SDF with fluoride varnish in comparison with an established, active comparator of glass ionomer sealants and atraumatic restorative treatment with fluoride varnish for dental caries. A total of 60 schools were originally proposed to be enrolled. The study used a 2-stage enrollment process. First, eligible schools in the New York City area were solicited for participation. Inclusion criteria for school enrollment included an overall student population of 80% or higher receiving free or reduced-cost lunch and at least 50% of enrolled students reporting Hispanic or Latino or Latina ethnicity and/or Black race. Second, informational letters and informed consent documents were distributed to all children enrolled in participating schools.

Exclusion criteria for individual participants included any child who did not speak English and children enrolled in special education classrooms. For ethical purposes, any child in an enrolled school was eligible to receive care. However, only children in grades kindergarten through grade 3 were included in the study for analysis because they were expected to remain enrolled in the school at the time of follow-up. Due to contractual obligations with the New York State Department of Health, data could only be collected from children if they were still enrolled in the included schools.

### Randomization

Consenting schools were listed in ascending order of population size and block randomized in blocks of 4 schools using a 1:1 allocation ratio. Allocation sequences were created using a random number generator.^22^ Allocation was performed at the school level and concealed from the potential participants within each school. Randomization was performed by R.R.R. and verified by T.B.-G.

### Interventions and Procedures

Children were randomized at the school level to receive either an experimental condition or standard of care active comparator treatment. The experimental treatment consisted of 5% fluoride varnish applied to all teeth and 38% SDF (2.24 mg/dose of fluoride ion) applied to all asymptomatic cavitated lesions and brushed on all pits and fissures of bicuspids and molars. The standard of care treatment included identical application of fluoride varnish, glass ionomer sealants applied to all pits and fissures of bicuspids and molars, and placement of atraumatic restorations on all frank asymptomatic cavitated lesions.

Treatments were provided in a single application after a baseline examination. For the experimental treatment, a single drop of 38% SDF was dispensed into a disposable mixing well and applied as specified for a minimum of 30 seconds. Treated sites were then air dried for a minimum of 60 seconds. For standard of care, a cavity conditioner was applied to pits and fissures for 10 seconds. Glass ionomer sealant capsules were mixed for 10 seconds at 4000 revolutions per minute and then applied directly via the finger-sweep technique and digitally applied to all pits and fissures, ensuring that closed margins were achieved. All treatments were provided in a dedicated room in each school using mobile equipment by dental hygienists or registered nurses with the support of assistants and under the supervision of a licensed dentist. No personalization of the treatment plan was required or performed.

### Outcomes

At each observation, standardized study clinicians performed full-mouth visual-tactile oral examinations. Teeth were assessed as being present or missing intraorally. Caries diagnosis was performed using the standard International Caries Detection and Assessment System (ICDAS II) adapted criteria for epidemiology and clinical research settings.^23^ Individual tooth surfaces were assessed as being intact or sound (ICDAS II codes 0-4), sealed, restored, decayed (ICDAS II codes 5-6), or arrested.

Our primary outcomes were the proportion of children with arrested carious lesions (arrest) and the proportion of children with no cumulative incidence of decayed teeth from previously sound dentition (prevention). Arrest failure was recorded if the tooth presented at baseline with untreated caries, received treatment at baseline with either experimental treatment or standard of care, and presented at follow-up with either untreated caries or a filling (indicative of treatment for caries applied by an external clinician). If a tooth was exfoliated prior to the 2-year follow-up that was coded as arrested after baseline treatment, that tooth was discounted from analysis. Caries prevention compared the cumulative incidence of caries in each treatment group. Children with new caries included those who presented at follow-up with either (1) untreated carious lesions or (2) presence of a filling not present at baseline. The decay determination was previously used in assessing the effectiveness of the active comparator.^24,25^

Outcomes were aggregated at the individual level to mitigate within-individual correlation for participants having multiple lesions at baseline or multiple new caries at follow-up. If a child at baseline presented with multiple carious lesions that received treatment, a failure of any treated lesion at follow-up was considered person-level arrest failure regardless of the status of other lesions. Similarly, caries incidence was considered prevention failure regardless of how many lesions were observed.

Other outcomes of the CariedAway trial not reported here include the 4-year prevention rate of caries, the noninferiority of registered nurses vs dental hygienists in the effectiveness of treatment with SDF,^26^ and effects on oral health-related quality of life,^27^ academic performance, and school absenteeism.

### Demographic Variables

Demographic data (including age, sex, and race and ethnicity) were self-reported by parents or guardians on informed consent documents. Selectable options for race and ethnicity were the same as those required by the New York City Department of Education, including American Indian or Alaskan Native, Asian, Black or African American, Hawaiian or Pacific Islander, and White. An “Other” option was provided that was to include any other race not listed. Ethnicity options included Hispanic (Latino or Latina) or non-Hispanic. Race and ethnicity data were collected to ensure that the targeted study population was included and for future stratified analyses.

### Blinding

Participants were blinded to their group assignments; however, given the staining effect of SDF on untreated decay, it was possible that patients could derive their treatment assignment. Clinicians and examiners were not blinded due to the specific procedures required for each treatment.

### Statistical Analysis

Statistical analysis is reported through March 2022. Our approach for noninferiority followed established guidelines.^28^ Power analyses for primary clinical outcomes was calculated for a 2-group clustered trial design and previously reported (N = 396).^21^ The intraclass correlation for dependence within cluster was estimated via mixed-effects multilevel logistic models.

Our noninferiority margin was predetermined to be 10% as the maximum clinically relevant difference and also agrees with the fixed margin method when comparing our active control with a placebo, in which prior investigations showed a prevalence of pit or fissure dentin caries of 1.6% vs 4.6% for dental sealants vs placebo and a risk reduction of 10% among children receiving sealants and atraumatic restorative treatment.^24,25^ For analysis of the proportion of children with arrested caries or no caries incidence in active control (*C*) and SDF (*S*) treatments, our null hypothesis was thus *H*_0_: *C* − *S* ≥ 10, while our alternative hypothesis was *H_a_*: *C* − *S* *<* 10, where 10 represents the selected noninferiority margin. Our statistical test for this hypothesis used 2-sample proportion tests, adjusting for any clustering effect of schools and comparing the upper bound of the 2-sided 95% CI for (*C* − *S*) with the noninferiority margin.^28,29^ As a sensitivity analysis, we performed similar tests using bootstrapped 95% CIs with schools as the cluster unit and 10 000 replications.

Intention-to-treat analysis was performed using multiple imputation. Five imputed data sets were generated for the full follow-up sample (N = 2998). Imputed data sets were then separated for arrest and prevention outcomes, following primary analysis procedures, and analyzed using logistic regression. Analysis was conducted in Stata, version 17 (StataCorp LLC) and R, version 1.4 (R Group for Statistical Computing). All *P* values were from 1-sided tests and results were deemed statistically significant at *P* < .025.

## Results

A total of 2998 children (1566 girls [52.2%] and 1432 boys [47.8%]; mean [SD] age at baseline, 6.6 [1.2] years) were recruited and treated. A total of 314 children (10.5%) presented at baseline with preexisting dental sealants on any tooth, and 874 (29.2%) had untreated dental caries (Table 1). Hispanic or Latino and Black children comprised 63.8% of the analytic sample (887 of 1390). The mean (SD) time that elapsed from baseline to follow-up for the analytic sample was 718 (87.2) days. The intraclass correlation coefficient was 0.034 for caries arrest and 0.0031 for caries prevention.

**Table 1.** Baseline Sample Description Overall and by Treatment Group

Characteristic	Full sample, No. (%)			Follow-up sample, No. (%)		
	All (N = 2998)	Experimental group (n = 1554 [51.8%])	Control group (n = 1444 [48.2%])	All (N = 1398)	Experimental group (n = 611 [43.7%])	Control group (n = 787 [56.3%])
Girls	1566 (52.2)	786 (50.6)	780 (54.0)	753 (53.9)	321 (52.5)	432 (54.9)
Boys	1432 (47.8)	768 (49.4)	664 (46.0)	645 (46.1)	290 (47.5)	355 (45.1)
Race and ethnicity						
Asian	36 (1.2)	20 (1.3)	16 (1.1)	24 (1.7)	14 (2.3)	10 (1.3)
Black	456 (15.2)	249 (16.0)	207 (14.3)	208 (14.9)	98 (16.0)	110 (14.0)
Hispanic	1397 (46.6)	685 (44.1)	712 (49.3)	679 (48.6)	287 (47.0)	392 (49.8)
Multiple	58 (1.9)	34 (2.2)	24 (1.7)	20 (1.4)	8 (1.3)	12 (1.5)
White	75 (2.5)	38 (2.4)	37 (2.6)	29 (2.1)	17 (2.8)	12 (1.5)
Other^a^	22 (0.7)	14 (0.9)	8 (0.6)	11 (0.8)	7 (1.1)	4 (0.5)
Missing	954 (31.8)	514 (33.1)	440 (30.5)	427 (30.5)	180 (29.5)	247 (31.4)
Age at baseline, mean (SD), y	6.6 (1.2)	6.6 (1.3)	6.7 (1.2)	6.6 (1.2)	6.6 (1.2)	6.7 (1.2)
Untreated decay	874 (29.2)	482 (31.0)	392 (27.1)	413 (29.5)	193 (31.6)	220 (28.0)
Sealants at baseline	314 (10.5)	144 (9.3)	170 (11.8)	156 (11.2)	60 (9.8)	96 (12.2)
Decayed teeth, mean (SD)	0.7 (1.4)	0.7 (1.5)	0.6 (1.4)	0.7 (1.4)	0.7 (1.4)	0.7 (1.4)

^a^ Other includes any other race not listed, including American Indian or Alaskan Native and Hawaiian or Pacific Islander.

**Figure 1.**
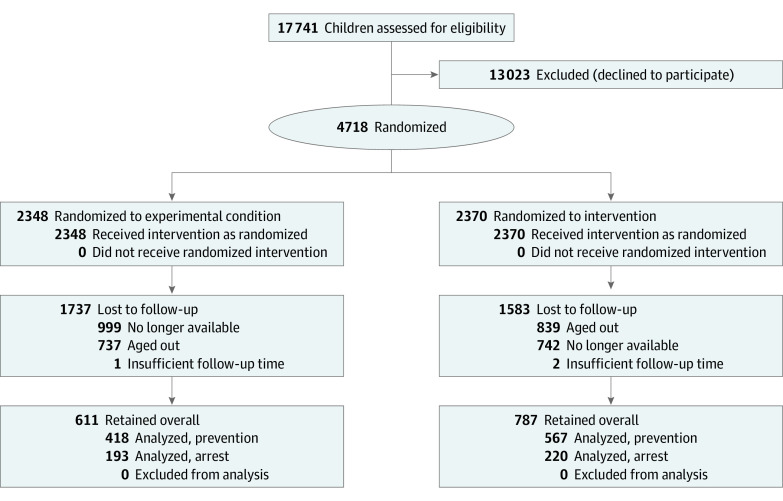
Study Flow Diagram

Our analytic sample consisted of all children in kindergarten through grade 3 who were enrolled, randomized, and treated and who completed a follow-up visit after approximately 2 years. A total of 4718 children across 47 schools were treated at baseline between September 16, 2019, and March 12, 2020, prior to suspension due to COVID-19 (Figure 1). As all children in schools were eligible for care, a subset of these participants were treated for ethical reasons but were outside of analytic grades, including children in grades 4 and 5 who would not be enrolled in school long enough to undergo follow-up. When restricted to children in viable grades, the enrolled and treated sample was 2998. We completed follow-up observations between June 7, 2021 and March 2, 2022, with 1398 children (611 in the experimental group, 20.4% of the enrolled and treated sample of 2998 children; 787 in the active control group, 26.3% of the enrolled and treated sample of 2998 children), for an overall follow-up rate of 29.6% (1398 of 4718) among all children enrolled and 46.6% (1398 of 2998) among all viable participants. As caries arrest can be evaluated only in children who had untreated decay at baseline, the analytic sample for arrest was 413 patients. The analytic sample for prevention was 985 patients. There were no adverse events reported.

**Figure 2.**
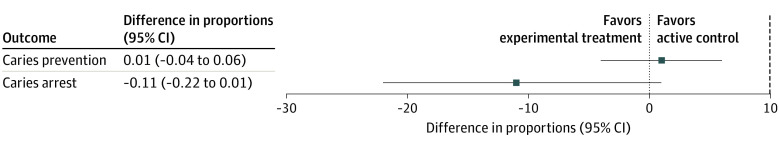
Noninferiority Plot for Caries Arrest and Prevention at 2 Years Whiskers indicate the 95% CI comparing the experimental group with the active control for caries arrest and prevention. The dashed line denotes the noninferiority limit. If the right-sided 95% CI does not exceed this threshold, then noninferiority is determined. This is equivalent to a 1-sided test.

The mean (SE) proportion of children with all caries remaining arrested was 0.56 (0.04) in the experimental group and 0.46 (0.04) in the control group, for a difference of −0.11 (95% CI, −0.22 to 0.01) (Table 2). The mean (SE) proportion of children without caries at baseline who remained caries free at follow-up was 0.81 (0.02) in the experimental group and 0.82 (0.02) in the control group, for a difference of 0.01 (95% CI, −0.04 to 0.06). Results from analyses using bootstrapped 95% CIs were not appreciably different for either caries arrest (difference between groups, −0.11; 95% CI, –0.27 to 0.002) or caries prevention (difference between groups, 0.01; 95% CI, −0.04 to 0.06). Experimental group rates were noninferior to those of the active control. Noninferiority for clinical outcomes is summarized in Figure 2.

**Table 2.** Noninferiority Results for Caries Arrest and Prevention After 2 Years

Outcome	Experimental group			Control group			Difference			
	No. (mean)	SD	SE	No. (mean)	SD	SE	No. (mean)	SD	SE	95% CI
Caries arrest	193 (0.56)	0.50	0.04	220 (0.46)	0.50	0.04	413 (–0.11)	1.24	0.06	–0.22 to 0.01
Caries prevention	418 (0.81)	0.39	0.02	567 (0.82)	0.39	0.02	985 (0.01)	0.78	0.03	–0.04 to 0.06

With imputed data for children with caries arrest (n = 874), the estimated control proportion of caries arrest was 0.47, yielding a corresponding odds ratio (OR) noninferiority margin of approximately 0.6. Comparisons of the estimated treatment effect OR to 1/OR noninferiority margin indicates that the experimental treatment remained noninferior for caries arrest (OR, 1.49; 95% CI, 0.91-2.44) (Table 3). With imputed data for children with caries prevention (n = 2124), the estimated active control proportion of prevention was 0.81, for a corresponding OR noninferiority margin of 0.55. The estimated effect for prevention (OR, 0.93; 95% CI, 0.68-1.27) was similarly noninferior.

**Table 3.** Noninferiority Results for Caries Arrest and Prevention After 2 Years, Imputed

Outcome	No.	Odds ratio^a^	SE	*t* Value	*P* value	95% CI
Caries arrest	874	1.49	0.33	1.79	.10	0.91-2.44
Caries prevention	2124	0.93	0.14	–0.51	.62	0.68-1.27

^a^ Odds ratio estimates compare experimental vs active control for each outcome.

## Discussion

In this randomized clinical trial of SDF vs dental sealants, an experimental treatment of SDF and fluoride varnish was noninferior in the 2-year arrest and prevention of dental caries compared with a standard active comparator, a package of glass ionomer sealants and atraumatic restorations and fluoride varnish. The arrest rate for the experimental treatment was considerably higher than for the active control, and the upper bound for the arrest difference of 0.01 nearly demonstrated superiority.

Without proper and timely intervention, dental caries and other oral diseases can lead to severe systemic infections,^30^ may negatively affect oral health-related quality of life,^31^ and are associated with decreased student academic performance and school attendance.^32^ To address the high rate of untreated caries in high-risk populations, the Centers for Disease Control and Prevention recommends school-based sealant programs, which have demonstrated clinical effectiveness and cost effectiveness.^33,34,35^ Our results potentially support the use of SDF as an arresting and preventive agent for school-based oral health programs and complement previous findings from other studies of nonrestorative treatments in schools.^24^

Overall, we showed that SDF and sealants had an approximate 80% caries prevention rate and 50% caries arrest rate after 2 years. These findings are comparable to those from other more controlled clinical studies, which indicated no differences in the 6- and 12-month caries arrest rates comparing SDF vs atraumatic restorative treatment.^19^ In addition, a prior review on the effect of SDF in preventing caries in primary dentition showed significant reductions in the development of new caries vs placebo after 24 months and was not more or less effective after 12 months compared with glass ionomer sealants.^15^ Our randomized design and ethnically diverse student population supports the generalizability of results to urban primary schools.

### Limitations

This study has some limitations. Our analysis classified each study participant as positive or negative for caries prevention or arrest and thus did not distinguish between single tooth failure and multiple tooth failures. This classification was done to ensure that the comparison for study outcomes was conservative, wherein any instance of failure at the tooth level would be considered failure at the individual level, regardless of how many failures were actually observed, and is in accordance with prior studies of school-based caries prevention.^24^ Future analyses of CariedAway incorporating longer periods of follow-up will subsequently consider alternative definitions of failure, analyzing the rate of failure at both the tooth and surface levels. These analyses will also provide data on whether the severity of baseline decay is a moderator in the overall effectiveness of treatment.

The approximate 2-year gap between initial treatment and follow-up coincided with municipal policies stemming from COVID-19 infection rates in New York City, with baseline observations being conducted over a 6-month period from September 2019 to March 2020. On March 16, 2020, schools were closed citywide, and dental offices suspended care except for emergency procedures. Schools remained closed to all school-based health programs throughout the 2020-2021 academic year. The original study protocol stipulated that children would be followed up biannually, but the resulting gap in observation from baseline to first follow-up was 2 years. Although our analysis of primary outcomes for caries arrest and prevention at 2 years was not disrupted, the gap in observation meant that treated teeth could be exfoliated prior to follow-up and thus could not be included in analysis. In addition, only 47 of the originally proposed 60 schools were enrolled. This resulted in slight differences in total treatment group enrollment and baseline attributes; however, as the study analyzed caries arrest and prevention in isolation, these concerns are attenuated. Furthermore, preliminary power calculations for CariedAway estimated a necessary sample size of 396 that we artificially inflated by an a priori assumption of an intraclass correlation coefficient of 0.10, reflecting a moderate expectation of cluster correlation.^21^ As we have shown, the actual degree of cluster correlation within schools is negligible. As a result, differences in the total study population should not have an appreciable effect on power.

Due to the continued effect of COVID-19, our follow-up rates among viable enrolled children was 46.6% (1398 of 2998). To partially address this limitation, we supplemented our original analysis with multiple imputation, and the results in the imputed sample were not different from those of the sample with completed follow-up observations. Despite this finding, our results should be interpreted with caution. Future longitudinal analysis of the CariedAway data will use all available observations of enrolled children to further expand on the presented analysis.

New York City dental offices were authorized to reopen in June 2020 after the adoption of interim infection control and prevention guidelines, specifically the reduction of aerosol-generating procedures. Due to these restrictions on preventive care, combined with the CariedAway population being specifically chosen because of their traditional lack of access to or use of routine dental care, it is unlikely that confounding dental treatments were received in the time between observations.^36^ We further attempted to adjust for this in the analysis of caries arrest and prevention by considering both untreated decay and any new fillings that were not present at baseline, which would be indicative of new disease incidence prior to follow-up.

## Conclusions

The benefits of the caries arrest and prevention methods tested in CariedAwa*y* offer opportunity for expanding access to critical oral health care worldwide. As school-based dental sealant programs are limited by burdening costs and lack of available, trained clinicians,^17^ use of SDF may offer an attractive alternative approach to school-based caries prevention.

## Notes

Supplement 1.
Trial Protocol

Supplement 2.
Data Sharing Statement
