# Supplementary material for: Effect of Silver Diamine Fluoride on Caries Arrest and Prevention: The CariedAway School-Based Randomized Clinical Trial
Source: JAMA Netw Open. 2023 Feb 9;6(2):e2255458. doi: 10.1001/jamanetworkopen.2022.55458 (PMC9912124; doi:10.1001/jamanetworkopen.2022.55458)
Supplement: Supplement 1. — Trial Protocol [file jamanetwopen-e2255458-s001.pdf]

Comparative Effectiveness of School and Community-Based Caries Prevention Programs for Children in Underserved, Low Income, Hispanic Communities

This is a pragmatic, cluster, randomized controlled, non-inferiority trial which compares simple vs complex caries prevention. The study is being conducted in schools, at community-based locations, or at the New York University College of Dentistry. Simple is defined as fluoride varnish + silver diamine fluoride. Complex is defined as fluoride varnish + traditional sealants + interim therapeutic restorations (also referred to as temporary fillings). All interventions are: currently used in clinical practice; have American Dental Association billing codes; and, depending on the state and third party administrator, are compensated by Medicaid.

**Principal Investigator:**	Richard Niederman<br>Department of Epidemiology & Health Promotion<br>380 2nd Ave, Suite 301, 3rd Floor, New York, NY<br>212-998-9719<br>rniederman@nyu.edu
**Funding Support:**	Patient Centered Outcomes Research Institute<br>1828 L St., NW, Suite 900 Washington, DC 20036<br>Phone: (202) 827-7700 Fax: (202) 355-9558
**Study Intervention:**	The trial compares two caries prevention protocols delivered 1 time or 2 times each year in elementary schools, at community-based locations, or at the New York University College of Dentistry: a novel simple intervention of fluoride varnish + silver diamine fluoride vs the traditional complex intervention of fluoride varnish + traditional sealants + interim therapeutic restorations. 30 elementary schools will be randomized to receive simple prevention twice per year and 30 elementary schools will be randomized to receive complex prevention twice per year. The remaining 16 schools will receive simple prevention once per year.
**IRB Number:**	S17-00578

Initial version: V2 MAY 25, 2017
Amended: V3 July 27, 2018
Amended: V4 September 6, 2018
Amended: V5 January 14, 2019
Amended: V6 February 27, 2019
Amended: V7 August 13, 2019
Amended: V8 August 22, 2019
Amended: V9 September 12, 2019
Amended: V10 February 13, 2020
Amended: V11 October 19, 2020
Amended: V12 January 6, 2021
Amended: V13 April 13, 2021
Amended: V14 June 16, 2021
Amended: V15 December 15, 2021
Amended: V16 January 26, 2022
Amended: V17 April 1, 2022

Table of Contents

STUDY SUMMARY ... 6

1 INTRODUCTION ... 7

1.1 BACKGROUND AND SIGNIFICANCE ... 7
1.1.1 *Oral Health* ... *7*
1.1.2 *Education* ... *7*
1.1.3 *Need* ... *7*
1.1.4 *Geography* ... *7*
1.2 STUDY AIMS ... 7
1.3 INVESTIGATIONAL DEVICE ... 9
1.4 RESEARCH RISKS & BENEFITS ... 9
1.4.1 *Risk of Investigational Device* ... *9*
1.4.2 *Other Risks of Study Population* ... *9*
1.4.3 *Potential Benefits* ... *10*

2 STUDY OBJECTIVES ... 11

2.1 PRIMARY OBJECTIVE ... 11
2.2 SECONDARY OBJECTIVE(S) ... 12

3 STUDY DESIGN ... 12

3.1 GENERAL DESIGN ... 12
3.2 PRIMARY STUDY ENDPOINTS ... 16
3.3 SECONDARY STUDY ENDPOINTS ... 16
3.4 PRIMARY SAFETY ENDPOINTS ... 16

4 PARTICIPANT SELECTION, ENROLLMENT, AND WITHDRAWAL ... 17

4.1 STUDY POPULATION ... 17
4.2 PARTICIPANT RECRUITMENT, SCREENING AND ENROLLMENT ... 17
4.2.1 *Recruitment* ... *17*
4.2.2 *School Randomization* ... *18*
4.2.3 *School Rosters and Informed Consent Preparation* ... *18*
4.2.4 *Informed Consent* ... *19*
4.3 EARLY WITHDRAWAL OF PARTICIPANTS ... 20
4.4 DENTAL PROVIDER REFERRAL ... 20
4.4.1 *Children without a Dentist* ... *20*
4.4.2 *Children that require an urgent referral* ... *20*

5 STUDY DEVICE ... 21

5.1 DESCRIPTION ... 21
5.2 METHOD FOR ASSIGNING SUBJECTS TO TREATMENT GROUPS ... 23
5.3 IMPLANTATION OF STUDY DEVICE ... 23
5.4 SUBJECT COMPLIANCE MONITORING ... 23
5.5 PRIOR AND CONCOMITANT THERAPY ... 23
5.6 PACKAGING ... 23
5.7 BLINDING OF STUDY ... 23
5.8 RECEIVING, STORAGE, DISPENSING AND RETURN ... 23
5.8.1 *Receipt of Study Device* ... *23*

5.8.2 Storage ... 24
5.8.3 Dispensing ... 24
5.8.4 Return ... 24

6 STUDY PROCEDURES ... 24

6.1 DESCRIPTION ... 24
6.1.1 Informed Consent ... 24
6.1.2 Scheduling ... 26
6.1.3 COVID-19 Pre-Screening and Screening Procedures ... 26
6.1.4 Clinical Room Set-up ... 27
6.1.5 Waiting Area ... 28
6.1.6 Examination and Diagnosis ... 29
6.1.7 Prevention ... 29
6.1.8 Take Home Messages and Follow-on Care ... 29
6.2 PREVENTIVE CARE ... 30
6.2.1 Simple prevention ... 30
6.2.2 Complex prevention ... 30
6.3 STUDY ADMINISTRATION AND DURATION ... 31
6.4 CONTROL/COMPARISON GROUP AND CONFOUNDING FACTORS ... 32
6.5 RANDOMIZATION AND BLINDING ... 32
6.5.1 Randomization ... 32
6.5.2 Blinding ... 32
6.6 COMPLIANCE ASSESSMENT ... 32
6.7 PRIOR AND CONCOMITANT THERAPY ... 33
6.8 RECEIVING, STORAGE, TRANSPORTING, DISPENSING, BREAKDOWN AND DISPOSAL OF CLINICAL SUPPLIES ... 33
6.8.1 Receiving ... 33
6.8.2 Storage ... 33
6.8.3 Transporting ... 33
6.8.4 Dispensing ... 34
6.8.5 Breakdown ... 34
6.8.6 Disposal ... 34
6.9 STUDY PROCEDURES TABLE ... 35

7 STATISTICAL PLAN ... 36

7.1 SAMPLE SIZE DETERMINATION ... 36
7.2 STATISTICAL METHODS ... 36
7.3 SUBJECT POPULATION FOR ANALYSIS ... 37
7.4 INTERIM ANALYSIS ... 37

8 DATA AND SAFETY MONITORING PLAN (DSMP) ... 37

8.1 INFORMED CONSENT ... 37
8.2 RISK ... 38
8.3 IMPLEMENTATION ... 38
8.4 CONFIDENTIALITY ... 38
8.4.1 Protection of Subject Privacy ... 38
8.4.2 Confidentiality During Adverse Event (AE) Reporting ... 39
8.5 EXPECTED RISKS ... 39
8.6 ADVERSE EVENT/ UNANTICIPATED PROBLEMS ... 39
8.6.1 Definitions ... 39
8.7 TIME PERIOD AND FREQUENCY FOR EVENT ASSESSMENT AND FOLLOW-UP ... 40
8.8 CHARACTERISTICS OF AN ADVERSE EVENT ... 40

8.8.1 Relationship to Study Intervention *40*
8.8.2 Expectedness of SAEs *41*
8.8.3 Severity of Event *41*
8.9 REPORTING PROCEDURES 41
8.9.1 Reporting for Multi-Center Trials *41*
8.9.2 Unanticipated Problem Reporting *41*
8.9.3 Adverse Event Reporting of Non-IND Studies *42*
8.9.4 Adverse Event Reporting of Investigation New Drug (IND) Studies *42*
8.9.5 Events of Special Interest (if applicable) *42*
8.9.6 Reporting of Pregnancy *42*
8.10 HALTING RULES 42
8.11 QUALITY CONTROL AND QUALITY ASSURANCE 42
8.12 SUBJECT ACCRUAL AND COMPLIANCE 43
8.12.1 Measurement and Reporting of Subject Accrual *43*
8.12.2 Measurement and Reporting of Participant Adherence to Treatment Protocol ... *43*
8.13 STOPPING RULES 43
8.14 SAFETY REVIEW PLAN 43
8.15 SUBMISSION OF ON-SITE MONITORING/AUDIT AND INSPECTION REPORTS 44
8.16 DATA HANDLING AND RECORD KEEPING 44
8.17 DATA MANAGEMENT RESPONSIBILITIES 44
8.18 DATABASE PROTECTION 45
8.19 SOURCE DOCUMENTS/PROTECTIONS 45
8.20 CASE REPORT FORMS 45
8.21 RECORDS RETENTION AND STORAGE 45
8.22 STUDY/REGULATORY BINDER 45
8.23 SCHEDULE AND CONTENT OF REPORTS 45
8.24 INFORMED CONSENT 46
8.25 REPORTING CHANGES IN STUDY STATUS 47
9 ETHICAL CONSIDERATIONS 48
10 STUDY FINANCES 48
10.1 FUNDING SOURCE 48
10.2 CONFLICT OF INTEREST 48
10.3 PARTICIPANT STIPENDS OR PAYMENTS 48
11 PUBLICATION PLAN 48
12 ATTACHMENTS 48
13 REFERENCES 48

List of Abbreviations

- ADL = Activities of Daily Living
- *AE = Adverse Event*
- *BEDAC = Biostatistics and Epidemiology Data Analytics Center*
- *CDC = Centers for Disease Control*
- *COI = Conflict of Interest*
- *CRA = Clinical Research Associate*
- *CRF = Case Report Form*
- *CTSI = Clinical and Translational Science Institute*
- *DCC = Data Coordinating Center*
- *DSMB = Data and Safety Monitoring Board*
- *DSMP = Data Safety Monitoring Plan*
- *eICF = Electronic Informed Consent Form*
- *FDA = Food and Drug Administration*
- *GCP = Good Clinical Practice*
- GAMs = Generalized Additive Models
- HCP = Health Care Personnel
- *HEAL = Health Evaluation and Analytics Lab*
- *HIPPA = Health Insurance Portability Accountability Act*
- *IDs = Identifiers*
- *IND = Investigational New Drug*
- *IRB = Institutional Review Board*
- ML-MEM = Multilevel Mixed Effects Modeling
- MOU = Memorandum of Understanding
- *NESS = New England Survey Systems*
- *NHANES = National Health and Nutrition Examination Surveys*
- *NYC = New York City*
- *NYC DOE = Department of Education*
- *NYSDOH = New York State Department of Health*
- *NYU = New York University*
- *NYUCD = New York University College of Dentistry*
- *NYUMC = New York University Medical Center*
- *MOU = Memorandum of Understanding*
- *OHE = Oral Health Education*
- *OHRP = Office of Human Research Protections*
- *OSR = Office of Science & Research*
- *PCORI = Patient Centered Outcomes Research Institute*
- *PD = Professional Development*
- *PI = Principal Investigator*
- *PPE = Personal Protective Equipment*
- *QR = Quick Response Code*
- *REDCap = Research Electronic Data Capture*
- *UP = Unanticipated Problem*
- *SAE = Serious Adverse Event*
- *SDF = Silver Diamine Fluoride*
- *SOC = Standard of Care*

Study Summary

Title	Comparative Effectiveness of School and Community-Based Caries Prevention Programs for Children in Underserved, Low Income, Hispanic Communities
Short Title	CARIED*AWAY* NYC
IRB Number	S17-00578
Study Type	Human
Study Design	Pragmatic, cluster, randomized controlled, non-inferiority trial
Study Intervention	Simple prevention (fluoride varnish + silver diamine fluoride) vs complex prevention (fluoride varnish + traditional sealants + interim therapeutic restorations)
Study Duration	5 years
Study Location(s)	The study will be conducted in New York City Title 1 Elementary Schools, community-based locations, or at the New York University College of Dentistry
Primary Objective	To determine if the newer simple caries prevention is not inferior to the traditional complex caries prevention
Sample Size	76 schools (approximately 20,000 children)
Diagnosis and Main Inclusion Criteria	The disease/condition is untreated caries. All children in the participating schools with informed consent will be included. The only exclusion criteria are children not in the participating schools, children without informed consent and children that are allergic to silver.
Study Product and Planned Use	Silver diamine fluoride (Advantage Silver Arrest*).* The liquid is administered topically to carious lesions, as well as pits and fissures of posterior teeth.
Control / Comparison	The new simple prevention will be compared to the traditional complex prevention.
Statistical Methodology	Primary outcomes include caries arrest, caries prevention, and quality of life. We will assess these using multilevel binomial regression and generalized estimating equations.

1 Introduction

This study will be conducted in compliance with the protocol approved by the Institutional Review Board (IRB), Good Clinical Practice (GCP) guidelines, and applicable New York University Medical Center (NYUMC) and federal regulatory requirements.

1.1 Background and Significance

1.1.1 Oral Health

More than 50% of U.S. elementary school-age children have experienced caries (tooth decay or cavities), and more than 20% have untreated caries[1]. The oral health needs of low-income minority children are almost double U.S. averages – current estimates indicate that 70% have experienced caries, 38% have untreated caries, 66% do not have sealants, and all have difficulty accessing care[1.] Even more troubling, children with untreated caries have an incidence of sepsis ranging from 5% to 10%[2]. In rare instances, untreated decay can lead to serious systemic infections and even death[3]. Currently, only 25% of children in New York City (NYC) schools receive school-based oral health care. Importantly, these school-based oral health programs all vary in service provision and scope, and none have outcome measures to assess health equity.

1.1.2 Education

Oral health has larger connections to general health and child development, such as academic performance and quality of life. Oral health also affects children's education. Children with untreated caries and associated toothaches have more school absences, cannot pay attention in school, do not keep up with their peers academically, and have lower standardized test scores4[-8.] Additionally, children who require dental care in a traditional dental office frequently miss school.

1.1.3 Need

Based on 3 years of conversations, surveys, and guidance, parents and stakeholder partners in New York City requested school-based caries prevention[9-10], and tracking the following patient-centered outcomes: untreated cavities, quality of life, and school performance. Further, these parent and stakeholder partners participated in planning, designing, and piloting the proposed study. These partners are also participating in the proposed study's conduct, dissemination, and implementation.

1.1.4 Geography

NYC elementary schools serve over one million children of varying socioeconomic and racial/ethnic backgrounds, many of which are from those demographic groups with the highest oral health needs. This initiative focuses on improving the oral health of NYC children. We will enroll 76 schools across NYC to receive alternative caries prevention treatments.

1.2 Study Aims

The overall goal of this study is to measurably improve oral health equity. Towards this goal, we will compare "simple" and "complex" prevention, two packages of interventions designed to prevent and arrest dental caries. The study design is a cluster randomized non-inferiority pragmatic trial. Both preventive interventions are expected to be equally effective in improving health equity. The major difference in the two protocols is that "simple" prevention costs

approximately 25% of “complex” prevention. Simple also takes approximately 25% of the time of complex prevention. Therefore, if simple and complex are found to be equally effective, for the same time and cost, simple prevention could improve health and health equity to approximately 3 times the number of children as complex prevention.

Our prior work, and data from systematic reviews, indicates that the protocols we propose should be similarly effective in both arresting current caries and preventing subsequent caries by 50% to 80%. Thus all children should benefit from the proposed preventions. We will collect data on oral health and child quality of life, and assess student academic performance.

The study consists of three groups: (1) a group receiving simple prevention twice per year; (2) a group receiving complex prevention twice per year; and (3) a group receiving simple prevention once per year.

We will select 76 elementary schools to participate in the program over a five-year period. 30 elementary schools will be randomized to receive simple prevention twice per year and 30 elementary schools will be randomized to receive complex prevention twice per year. The remaining 16 schools will receive simple prevention once per year. The care will be provided in schools, community-based locations or at the New York University College of Dentistry.

The inclusion criteria for the schools enrolled in groups (1) and (2) include: A Hispanic/Latino population of at least 50% and a free and reduced lunch population of at least 80%. The remaining racial/ethnic and socioeconomic distribution of these schools will include children from any race/ethnicity and economic background. The inclusion criteria for schools in group (3) include any elementary school in New York City and will be selected to include representation from every major racial/ethnic group and socioeconomic level.

For the purposes of the analysis on dental-related service use and costs among Medicaid beneficiaries, all children participating in Medicaid of elementary school age will be included in the analysis, except for children in participating schools that do not provide informed consent to provide oral health and Medicaid dental service use data.

The Primary Specific Aims are to:

1. Determine the non-inferiority of simple vs complex caries prevention for preventing new caries (primary prevention).
2. Determine the non-inferiority of simple vs complex caries prevention for arresting current caries (secondary prevention).
3. Determine the impact of simple vs complex prevention on child oral health-related quality of life
4. Determine the impact of once per year vs twice per year care on the arrest and prevention of caries

Notably, toothaches or office based dental care reduces in-class learning time. If the caries prevention programs are as effective as predicted, knowing the time/trade-off will be important. Thus a secondary outcome of the study will be school performance (school absence and student achievement). To insure accurate assessments, program participants will be compared to children in their own schools and to children in NYC “peer schools” not participating in the study. No additional data is collected for educational outcomes from participants.

The Secondary Specific Aims are to:

1. Determine the impact of prevention vs no prevention on school absence
2. Determine the impact of prevention vs no prevention on standardized test performance
3. Determine the impact of simple vs complex prevention on school absence and test performance

The exclusion criteria will be those: (1) not attending participating schools, (2) those attending participating schools without informed consent, or (3) those attending participating schools with informed consent, but without assent and (4) those allergic to silver.

For the purposes of the analysis on dental-related service use and costs among Medicaid beneficiaries, the only excluded groups are (1) children not participating in Medicaid will be excluded and (2) children participating in Medicaid in Caried*Away* schools, but without informed consent to provide oral health and Medicaid dental service use data.

1.3 Investigational Device

Silver diamine fluoride (SDF) is a **Category B low risk device**. It is a clear liquid, used for topical oral application on sensitive dentinal lesions to reduce sensitivity; on carious lesions to arrest decay; and on pits and fissures to prevent caries. Application is eligible for Medicaid coverage in some states.

1.4 Research Risks & Benefits

1.4.1 Risk of Investigational Device

The only known risks are allergies to silver and short-term (3-7 day) irritation from dripping on gingiva or epithelium. However oral health professionals working in this study will be trained on the proper techniques to avoid risks. All students with a silver allergy will be excluded from participating. The application of silver diamine fluoride may discolor any cavities resulting in a brown or black color. This color change indicates that the cavity has stopped growing. If accidental skin contact occurs it can cause a temporary light brown staining to the lips, cheeks, or permanent staining to clothing. For stain removal to the skin, soap and water are applied immediately. The same procedure is applied for cleaning clothes.

1.4.2 Other Risks of Study Population

1.4.2.1 Fluoride varnish

Children that have a nut allergy. Some fluoride varnish products contain pine nuts as an ingredient in their varnish resin formula. There are 2 flouuride varnish products being used in this study. The first product is called FluoroDose® and **will not** contain pine nuts as an ingredient. This product will be used for children that have a nut allergy. The second fluoride varnish product is called PreviDent® and will be used on children that do not have a nut allergy. These products are listed in section 5 under Study Device.

1.4.2.2 COVID-19

Care follows WHO guidance for essential, aerosol-free care. 2. The care is standard of care, based on American Dental Association guidelines. 3. Care

delivery follows current CDC guidelines for PPE. 4. Our subject population does not include categories of individuals currently known to be at greater risk of serious COVID-19 consequences.

In school and community-based programs have unique characteristics that warrant specific infection prevention considerations due to risk for exposure to COVID-19. **Enhanced precautions** are in place to avoid transmission of SARS Cov-2 in school and community-based settings.

All New York University College of Dentistry Health Care Personnel (NYUCD HCP) are required to get COVID-19 diagnostic PCR tests within 14 days prior to returning to work and submit verification, be tested every 14 days and complete the NYU daily screener. Any NYUCD HCP who fails on the “screener pass” will automatically be referred to the NYU COVID prevention & response team and will not report to work.

All clinical staff will wear personal protective equipment consistent with CDC, DOH, FDA and NYU Dentistry guidelines.

NYUCD HCP will be conducting all study related procedures at all locations. NYUCD HCP will wear PPE based on current NYU Dentistry guidelines. PPE is listed below:

1. Disposable gowns
2. Disposable gloves
3. N95 respirator that have been fit tested for a specific respirator and shall be disposed of at the end of each clinical day
4. Level 3 surgical masks
5. Goggles
6. Face Shields
7. Bonnets
8. Booties

Gloves are changed between patients. Goggles and face shields are disinfected with an EPA registered intermediate level surface disinfectant between patients. All dental chairs, stools and touch surfaces are disinfected with an EPA registered intermediate level surface disinfectant at the beginning of each day, prior to and after treating patients, and at the end of the day.

All NYUCD HCP including supervisory professionals will ensure that care is provided and overseen in a way that maximizes benefits while avoiding harm to personnel, students and parents/guardians from potential exposure to COVID-19 infection.

1.4.3 Potential Benefits

There are three distinct benefits.

1. The benefit to all participants is that they will receive either primary or primary and secondary caries prevention once or twice yearly. The expectation is that both the simple and complex will reduce caries by 50% to 80%.

2. If, as we propose, simple prevention is as effective as complex, and simple prevention takes approximately ¼ the time and ¼ the cost of complex prevention, its use will increase access to effective care.
3. The methods to be applied and the outcomes identified both address national Healthy People 2020 goals.

Ultimately, evidence-based changes in knowledge, attitudes and practice among all patient and stakeholder partners will help reduce the continually high burden of caries in children, particularly among minority and low-SES children. Improved outcomes data should increase support for the sustainability of ongoing and new caries prevention programs at both a local and national level. The benefits to society include improved oral health for our children that is sustained by a system of preventive care that is more clinically and cost effective than the current method of care.

Numerous efficacy trials demonstrate the benefits of complex preventive dental care, which has far-reaching effects on physical and emotional health, nutrition, social interactions and employability. However, numerous barriers still exist to improvements in child oral health: an already-existing global and national burden of caries; the burden of disease in NYC; the social burden of disease, particularly affecting academic performance and psycho-social development; barriers to office-based care[9]; economic incentives to support treatment rather than prevention; the reduced efficacy of treatment to prevention; and large variation in care without outcome measures of effectiveness.

Without data to support the effectiveness of preventive oral health care, we predict that two things will occur (depending on state and local values and circumstances): 1) the current helter-skelter implementation of well-meaning but ineffective programs will continue; and/or 2) there will be an extinction of those public caries prevention programs that are truly effective.

From a national perspective, our preliminary data suggest that a school-based caries prevention program may represent the first program that exceeds the goals of *Healthy People 2000*. If this happened, it would be transformative. This information would: 1) set the stage for broader dissemination within NYC and other metropolitan areas; and 2) stimulate implementation tests of other “place-based” caries prevention programs for other populations (e.g.: mothers, elderly, etc.), in locations where people learn, work, play, and pray.

Long term, we believe that children (and adults) with improved oral health will be better prepared to learn, thrive and improve their social and economic opportunities. These important accomplishments would be one step in reducing social inequalities. The proposed school and community-based, cluster, randomized controlled trial, comparing two evidence-based caries prevention protocols, will address and potentially obviate all of the identified gaps.

2 Study Objectives

2.1 Primary Objective

To determine if simple prevention is non-inferior to complex prevention for caries arrest and longitudinal caries prevention.

2.2 Secondary Objective(s)

To compare the effectiveness of simple prevention when provided twice yearly versus once yearly.

To determine if children receiving either simple prevention or complex prevention will have reduced school absence and higher academic performance when compared to matched students in non-participating schools.

To determine if children receiving simple prevention or complex prevention will have reduced dental service use and costs in Medicaid when compared to matched students in non-participating schools.

3 Study Design

3.1 General Design

This is a pragmatic cluster randomized non-inferiority controlled trial comparing the clinical effectiveness of two caries prevention protocols. The study consists of three groups. Two groups will be randomly assigned at the school level to receive either simple or complex prevention provided twice yearly. A third group will be selected to receive simple prevention only, provided once yearly.

A total of 76 elementary schools will be selected from interested schools in New York City, identified via letters and phone calls to school principals. For groups (1) and (2) 30 elementary schools will be randomized to receive simple prevention twice per year and 30 elementary schools will be randomized to receive complex prevention twice per year. For group (3), 16 elementary schools will be selected to receive simple prevention once per year.

Beginning in the first year of the study, a total of 30 schools will be randomized (15 schools receiving simple prevention and 15 receiving complex prevention) to receive prevention twice per year consenting children will be followed over five years. In Year 2, 30 additional schools will be randomized to receive either simple or complex prevention twice per year. Consenting children will be followed over four years. Also in Year 2, an additional 16 schools will be selected to receive simple prevention once per year, and consenting children will be followed over four years. Starting in year 3, all care will be provided by NYUCD HCP, in school, at community-based locations, or at NYUCD. Starting in year 5, NYUCD licensed health professionals will continue to provide care and will also oversee all care provided by NYUCD senior dental hygiene students, in school, at community-based locations, or at NYUCD.

The inclusion criteria for the schools enrolled in groups (1) and (2) include: A Hispanic/Latino population of at least 50% and a free and reduced lunch population of at least 80%. The remaining racial/ethnic and socioeconomic of the schools beyond these criteria can include children from any race/ethnicity and economic background. The inclusion criteria for schools in group (3) include any elementary school in New York City. Schools in group (3) will be balanced to ensure representation from every primary race/ethnic group and from all socioeconomic tertiles.

The impact of COVID-19 has greatly impacted our in school-based cavity prevention approach. Elementary schools have closed, delayed in opening and will either allow students to return to

schools part-time under a blended learning model or will learn at home remotely full-time. This has impacted our ability to provide preventive services in some or possibly all of our schools. In order to continue to provide these services, our community engagement administrator will reach out to those schools where we cannot currently provide in school care, seek their guidance in helping to identify and connect with local community-based organizations in their area and then telephone these local organizations to discuss participation in the research study.

Once an alternate community-based location has been identified and is interested in participating, our HCP will meet with the community organization staff to review COVID-19 safety precautions at the community-based location, clinical protocols, room size and safe distancing requirements. NYUCD HCP will fill out the Caried*Away* COVID-19 community-based checklist. If the community-based location does not meet the requirements listed in the checklist, the NYUCD HCP will notify the Principal Investigator that the site is not an acceptable location for providing safe care.

If the community-based location meets the requirements of the Caried*Away COVID-19* community-based checklist and wishes to participate, the NYUCD HCP will review the Memorandum of Understanding (MOU). As community organizations are identified and sign an MOU we will submit their name and location to the IRB.

All clinical personnel will wear personal protective equipment consistent with CDC, DOH, FDA and NYU Dentistry guidelines.

NYUCD licensed health professionals will be providing care and will also oversee all care that is provided by NYUCD senior dental hygiene students at all locations. All clinical personnel providing care will wear PPE based on current NYU Dentistry guidelines. PPE is listed below:

1. Disposable gowns
2. Disposable gloves
3. N95 respirator that have been fit tested for a specific respirator and shall be disposed of at the end of each clinical day
4. Level 3 surgical masks
5. Goggles
6. Face Shields
7. Bonnets
8. Booties

Gloves are changed between patients. Goggles and face shields are disinfected between patients. All dental chairs, stools and touch surfaces are disinfected at the beginning of each day, prior to and after treating patients, and at the end of the day.

Caried*Away* school COVID-19 Checklist: The first day of the school visit the NYUCD HCP will review the school's policies for prevention of coronavirus transmission among students, staff and faculty using the Caried*Away* School COVID-19 Checklist. If there is any reason to believe that schools policies would put NYUCD HCP or students at risk, the NYUCD HCP Dental will contact the Principal Investigator to determine the appropriate course of action.

Consenting students in participating schools will receive care in a school, at a community-based location, or at NYUCD. Randomization will be done by computer and remain in effect during the duration of the trial. Care will be provided in a school, community-based locations or at NYUCD according to the group selection as described above. Participants in their first and second year

will contribute data to evaluate caries arrest. We will follow them longitudinally to evaluate prevention of new caries. The total expected school enrollment for the clinical and community components of the study is 76 schools.

Hypotheses and Interventions: We will test the primary hypothesis that school or community-based “simple” caries arrest and prevention provided twice yearly is equally effective (non-inferior) as school or community-based “complex” caries arrest and prevention provided twice yearly.

- “Simple” prevention is defined as fluoride varnish, silver diamine fluoride, fluoride toothpaste and oral hygiene instruction.
- “Complex” prevention is defined as traditional sealants, interim therapeutic restorations, fluoride varnish, fluoride toothpaste and oral hygiene instruction.

Table 1. Comparison simple and complex prevention on primary and secondary caries reduction*,**.

		Complex Prevention				Simple Prevention		
Focus:	Intervention:	Toothpaste	Fluoride Varnish	Sealant	Interim Therapeutic Restoration	Toothpaste	Fluoride Varnish	Silver Diamine Fluoride
1° Prevention	Smooth surface	25%[1]	40%[2]			25%[1]	40%[2]	
	Pits and fissure			80%[3]				80%[5]
2° Prevention	Arrest current caries				80%[4]			80%[5]

* Efficacy estimates of caries reduction from systematic reviews of human randomized controlled trials.
**References: 1.[11-12]; 2.[13]; 3.[14-16]; 4.[14, 17-19]; 5. [20-22]

Both types of care will be provided to all children with active informed consent in grades PK – 8 (ages 5 – 14). Participating schools will be randomized or selected at the school level to receive either simple or complex prevention. All participants in a given school, community-based location, or at NYUCD will receive the same prevention as randomized. At the beginning of each school year, all students in grades PK - 8 will be given a personalized pre-populated principal recruitment letter, the parent/guardian alternate location recruitment letter, the parent/guardian recruitment flyer, a health history, the NYU Langone Health COVID-19 research participation information sheet and a blank informed consent form.

Electronic informed consent forms will be distributed to parents/guardians of new students who have not previously consented to see if they wish to have their child participate. Electronic informed consents can be distributed by the schools using whichever existing method the school uses for mobile phone contact, in emails, or on the school's social media page in the following ways:

1. NYU Caried*Away* Website URL by selecting "registering your child"
   1) English
      - https://dental.nyu.edu/faculty/epidemiology-health-promotion/pcori-grant.html
      - Short URL: https://dental.nyu.edu/pcori
   2) Spanish
      - https://dental.nyu.edu/faculty/epidemiology-health-promotion/pcori-beca.html
2. Hyperlinks in English and Spanish
   - English language eICF: https://is.gd/CariedAwayNYC
   - Spanish language eICF: https://is.gd/CariedAwayNYC_SP
3. Caried*Away* email toolkit in English and Spanish

The parent or legal guardian fills out the eICF and uses 'scratch' method (mouse, stylus, or finger) to sign and then returns the completed ICF via REDCap.

The study administrator will then cross-reference the OSIS number in the original .csv file which was provided to us at the beginning of each school year by the NYC Department of Education, and verify that the correct OSIS number was provided correctly by the consenter.

The study administrator then uploads all of the informed consent PDF's to NESS via the secure and password protected ShareFile link listed below:

- https://nedental.sharefile.com

NESS then uploads each student's eICF to their electronic dental record in the iPad.

Examination, preventive care, and data collection by a proprietary secure electronic health record will be provided and collected up to 5 years, and all children in a given school will receive the same preventive care at the same frequency either at the school, at a community-based location or at NYUCD. Participants in their first and second years will contribute data to evaluate caries arrest, and then they will be followed longitudinally within schools. 50% of children $\geq$ 8 years old will be assessed with a validated quality of life instrument, and all children will receive a comprehensive oral examination at each visit. We will also test the secondary hypotheses that prevention: improves school performance and is equally effective given once per year as twice per year.

3.2 Primary Study Endpoints

Caries arrest and prevention will be determined at each visit. The primary end point for caries arrest will be at 2 years, and follow on to termination of trial determine the length of time that caries remains arrested. The primary end point for caries prevention will be at 3 years, and follow on to termination of trial to determine stability of prevention.

Presence / absence of caries is assessed by visual/tactile methods. Sound tooth surfaces appear white and smooth. Active caries appear brown and soft. Arrested caries appear dark brown or black and hard.

For clinical assessment, we will train and standardize clinicians using validated criteria implemented by the Centers for Disease Control and Prevention (CDC) in the National Health and Nutrition Examination Surveys (NHANES). Training and validation were developed and documented by our Colleague, Dr. Beltran, while at the CDC and field tested in the examination of special athletes[23,24]. Training and standardization of these examiners implement the World Health Organization guidelines[25,26].

3.3 Secondary Study Endpoints

School absence and academic performance data will be provided by the NYC Department of Education.

Medicaid claims, encounter, and eligibility data for elementary students in New York City enrolled in Medicaid will be provided by New York University's Wagner School of Public Service and New York University's School of Medicine.

3.4 Primary Safety Endpoints

The care provided is usual and customary care, not high-risk. The only risk about which we are aware is a toothache. Our history suggests that this occurs in approximately 1 in 2000 children. In our experience, this occurrence is far less than the percentage of children with tooth aches prior to initiation of prevention (approximately 5 in 100 children).

4 Participant Selection, Enrollment, and Withdrawal

4.1 Study Population

The study population will be children with informed consent who do not have an allergy to silver and are attending New York City elementary schools. 76 elementary schools will be selected to participate in the study. 60 of the participating schools will have at least 50% of the student population being of Hispanic/Latino origin and 80% from low socio-economic backgrounds. Beyond these inclusion criteria, any child in the school of any race/ethnicity or socio-economic group is eligible for participation. 16 of the 76 schools have no inclusion criteria but will be purposefully selected to represent a majority of students from every major racial/ethnic and socioeconomic group. School participation will be finalized with individual principals and we will execute a memorandum of understanding (MOU) with each participating school and each community-based organization.

4.2 Participant Recruitment, Screening and Enrollment

4.2.1 Recruitment

Recruitment will be done during Caried*Away* community engagement events and in concert with all other yearly school notifications and requests for consent via informational packets sent home with the children to their parents/guardians, mailed to the parents or sent electronically by the school's system of choice. Recruitment information will contain care details written in English and Spanish, a pre-populated personalized principal recruitment letter, the parent/guardian alternate location recruitment letter, the parent/guardian recruitment flyer, a health history, the NYU Langone Health COVID-19 research participation information sheet and a blank paper informed consent form. Parents will be asked to review the forms and contact the principal investigator if they have any questions prior to signing and returning the forms to the child's school.

Paper pre-populated personalized principal recruitment letter and blank informed consent will also be distributed and written in Arabic, Bengali Bangla, Chinese, French, Haitian Creole, Korean, Russian or Urdu.

Electronic informed consent forms will also be distributed to parents/guardians of new students who have not previously consented. The Caried*Away* parent/guardian communication toolkit emails contain hyperlinks to the NYU Caried*Away* Websites and REDCap eICF's in English and Spanish and will be provided to school administrators to distribute to parents and guardians of children enrolled in the school via whichever existing method the school uses for mobile phone contact, email or electronic communication systems.

The parent/guardian recruitment flyer (English and Spanish) will also be placed Independently during Caried*Away* community engagement events as an FAQ, as a flyer for posting on school bulletin board, as an attachment included in orientation documents for dental champions (school administrative staff, ie. (Parent coordinators) and dispersed to parent/guardian via whichever existing method the school uses for mobile phone contact, email or electronic communication systems, at the discretion of the school staff.

The English and Spanish parental/guardian recruitment flyers have the following Quick Response Code (QR code) in each flyer:

English QR code:

Spanish QR code:

The QR code allows the parent/guardian to use their smart phone to take a picture of the English or Spanish QR code. Once they have taken the picture they will be asked if they wish to Open “nyumc.org” in Safari. If they choose “open” they are sent directly to the IRB approved REDCap eICF in the English or Spanish. They can then securely fill out, sign and date the eICF.

All parents/guardians of new or previously consented students will be contacted via telephone by one of our clinical team members prior to scheduling any appointments. Parents/guardians of previously consented students will be reminded that they signed a 5 year informed consent. The clinical team member will use the research participant telephone recruitment script for COVID-19 updates. They will ask the parent/guardian a few questions to assess whether it makes sense, from a safety perspective due to the COVID-19 pandemic if they wish to have their child to participate or continue to participate in this research study. After a clinical team member speaks with the parent/guardian of a new participant, the clinical team member will send the parent/guardian the Caried*Away* new enrollee email written in English or Spanish. The email will also contain a link to the REDCap eICF and provide contact information if the parent/guardian has further questions.

4.2.2 School Randomization

Beginning in the first year of the study, a total of 30 schools will be randomized (15 schools receiving simple prevention and 15 receiving complex prevention) to receive prevention twice per year. Consenting children will be followed over five years. In Year 2, 30 additional schools will be randomized to receive either simple or complex prevention twice per year. Consenting children will be followed over four years. Also in Year 2, an additional 16 schools will be selected to receive simple prevention once per year, and consenting children will be followed over four years.

4.2.3 School Rosters and Informed Consent Preparation

In August we will request that the NYC Department of Education provide an electronic roster for each school. The rosters are used to create the IRB-approved pre-populated personalized principal recruitment letter from the principal to include school name, school address, and principal/assistant principal name and date explaining the program. The pre-populated personalized principal recruitment letters, the parent/guardian alternate location recruitment letter, the parent/guardian recruitment flyer, a health history, the NYU Langone Health COVID-19 research participant information sheet and a blank paper informed consent are printed at NYUCD and distributed to each teacher or school. These packets are either sent home with the children or mailed to the parent/guardian by the school administrator.

The REDCap electronic informed consent forms are updated by the study administrator annually and each time a new modification of the consent form has been approved by the NYU Langone IRB.

4.2.4 Informed Consent

Blank IRB-approved paper informed consent will be distributed during Caried*Away* community engagement events and to students at the beginning of each school year in concert with all other school notifications. Parents can also choose to sign their child up for the program at their child's school. The blank paper informed consent will include a pre-populated personalized principal recruitment letter from the principal, the parent/guardian alternate location recruitment letter, the parent/guardian recruitment flyer, program information, a health history, the NYU Langone Health COVID-19 research participant information sheet, and the blank informed consent to be filled out for signature by the parent/guardian.

Completed parent/guardian signed and dated paper informed consent will be returned to the school and collected by the research team. This protocol aligns with other school vision, hearing, and vaccination programs. Care will be provided to all children with informed consent.

Electronic informed consent forms will be distributed in addition to the established protocol for the distribution of paper consent forms to participating sites (schools). The following protocol will be followed for eICF distribution:

Electronic informed consent can be distributed by a clinical team member using the Caried*Away* new enrollee email written in English or Spanish using the following hyperlinks:

- English language eICF: https://is.gd/CariedAwayNYC
- Spanish language eICF: https://is.gd/CariedAwayNYC_SP

Electronic informed consent can be distributed by the schools in English or Spanish using whichever existing method the school uses for mobile phone contact, in emails, or on the school's social media page in the following ways:

1. NYU Caried*Away* Website URL by selecting "registering your child"
   1) English
      - https://dental.nyu.edu/faculty/epidemiology-health-promotion/pcori-grant.html
      - Short URL: https://dental.nyu.edu/pcori
   2) Spanish
      - https://dental.nyu.edu/faculty/epidemiology-health-promotion/pcori-beca.html
2. Hyperlinks in English and Spanish
   - English language eICF: https://is.gd/CariedAwayNYC
   - Spanish language eICF: https://is.gd/CariedAwayNYC_SP
3. CariedAway email toolkit in English and Spanish

The parent or legal guardian fills out the eICF and uses ‘scratch’ method (mouse, stylus, or finger) to sign and then returns the completed ICF via Redcap.

The study administrator will then cross-reference the OSIS number in the original .csv file which was provided to us at the beginning of each school year by the NYC Department of Education, and verify that the correct OSIS number was provided correctly by the consenter.

The study administrator then uploads all of the informed consent PDF’s to NESS via the secure and password protected ShareFile link listed below:

- https://nedental.sharefile.com

NESS then uploads each student’s eICF to their electronic dental record in the iPad

To facilitate accurate informed consent and student tracking we will request student rosters from the NY City Department of Education (NYC DOE). This will facilitate tracking students longitudinally. All forms will be translated into the major languages of NYC school children’s parents. Those languages are Arabic, Bengali Bangla, Chinese, English, French, Haitian Creole, Korean, Russian, Spanish and Urdu.

The consent form is valid for 5 years or as long as the child is in NYC schools. If guardianship of the child changes a new informed consent will need to be signed.

4.3 Early Withdrawal of Participants

A parent may withdraw their child at any time for any reason, with either a written request or a phone call (with verification) to the PI, CRA, or school. Upon withdrawal the center is notified, and the record flagged to remove the child from the informed consent list in the data base, in the Apple iPad electronic record. Students who withdraw are not replaced.

4.4 Dental provider referral

Parents of children without a dentist or children that require an urgent referral will be provided information on local dental providers in the following ways:

4.4.1 Children without a Dentist

Parents/guardians of children without a dentist can access the Caried*Away* Website Dentist finder map. They are instructed to click on the map, type in their address and then click on the blue pins to access the name, address and phone number for that provider. All providers listed accept new patients, see children and, accept Medicaid and CHIP. Parents will also be provided with the NYU CariedAway- addition to Website Dentist Provider Map Instructions.

4.4.2 Children that require an urgent referral

Parents/guardians of children that require an urgent referral will receive the NYU Caried*Away* parent communication urgent referral email sent to them by the Caried*Away* clinical mangers. This email provides them with the CariedAway- addition to Website Dentist Provider Map Instructions. The email also provides information for the NYU Department of Pediatric Dentistry.

5 Study Device

5.1 Description

We use three agents, all are off-label. None are investigational new drugs.: fluoride varnish (2 agents), and silver-diamine-fluoride. These agents are in common clinical use and are currently being provided by school-based oral health programs in NYC schools, have multiple randomized human clinical trials and systematic reviews indicating their efficacy in controlling caries, have American Dental Association billing codes, and are reimbursed by Medicaid and private insurers.
Manufacturer's information on each is provided below.

Fluoride Varnish

Proprietary Name: PreviDent® Varnish Mint, Grape and Raspberry

510(K) Number: K132109

Classification Name: Varnish, Cavity

Device Class: 2

Manufacturer: Colgate Oral Pharmaceuticals, 300 Park Avenue, New York, NY 10022

Indications for Use: PreviDent® Fluoride Varnish is a topical fluoride for the treatment of dentinal sensitivity.

Composition: PreviDent® 5% Sodium Fluoride Varnish contains 22,600 ppm fluoride. It has a strong desensitizing action when applied to dental surfaces, treating hypersensitivity quickly and easily. This product sets rapidly on contact with saliva, resulting in patient comfort and acceptance. PreviDent® Varnish will leave a thin film on the teeth after application.

Treatment Regimen:

1. Wash and dry tooth surface
2. Mix well prior to application
3. Apply product with supplied brush in the conventional manner
4. Apply a thin amount of varnish on the tooth's surface until the varnish surface is dry
5. Dispose of package and applicator after use

Post Application Instructions:

1. Hardens on contact with saliva so the patient may leave immediately after application of the product
2. It is recommended that the patient be instructed to eat only soft foods for 2 hours after treatment

Fluoride Varnish
Proprietary Name: FluoroDose®

510(K) Number: K982915

Classification Name: Varnish, Cavity

Device Class: 2

Manufacturer: Centrix, Inc., 770 River Road, Shelton, Connecticut, 06484

Indications for Use: FlouroDose® Fluoride Varnish is a topical fluoride for the treatment of dentinal sensitivity

Composition: FluoroDose® contains 5% Sodium Fluoride (NaF) or 22,600 ppm fluoride. FluoroDose is lactose, gluten free and **nut free**

Treatment Regimen:

1. Clean teeth and remove excess moisture from area to be treated
2. Grip the foil cover and slowly peel back, exposing the brush applicator and varnish well
3. Remove applicator brush and stir material in well
4. Paint a thin film of varnish onto treatment area
5. Let the varnish dry for approximately 10 seconds and then instruct the patient to close their mouth
6. Dispose of package and applicator after use

Post Application Instructions:

1. Patients may leave immediately after treatment
2. Patient should be advised to remain on a soft food diet and only drink cold liquids for two hours after treatment
3. Patients should be instructed not to brush for a minimum of 4 to 6 hrs.

Silver Diamine Fluoride

Proprietary Name: Diamine Silver Fluoride Dental Hypersensitivity Varnish

510(K) Number: K102973

Classification Name: Silver Dental Arrest

Device Class: 2

Manufacturer: Elevate Oral Care, 346 Pike Road, Suite 5, West Palm Beach, Florida 33411

Indications for Use: Silver diamine fluoride is a clear liquid. It is applied to sensitive teeth, carious lesions, and pits and fissures using Advantage Arrest Applicators. The concentration of fluoride in SDF and fluoride varnish are indicated in the table below:

Fluoride product	Unit dose (ml)	Concentration (ppm)	F ion mg/ml	F ion mg/dose
SDF 38%	1 drop (0.05)	44,800	44.8	2.24
FV 5% NaF	0.25	22,600	22.6	5.65
	0.4	22,600	22.6	9.04
	0.5	22,600	22.6	11.3

Composition:

Desensitizing Ingredient: Aqueous Silver Diamine Fluoride, 38.3% to 43.2% w/v

Inactive Ingredients: Purified water, FD&C Blue 1

Treatment Regimen:

1. Isolate the affected area of the tooth with cotton rolls
2. Clean and dry the affected tooth surface
3. Dispense 1-2 drops of solution into a dappen dish
4. Transfer material directly to the tooth surface with an advantage arrest applicator brush for approximately 30 seconds

5. Allow to dry, do not rinse

Post Application Instructions:

1. If accidental contact with skin or clothing occurs gently apply immediately with soap and water

5.2 Method for Assigning Subjects to Treatment Groups

This is a cluster randomized controlled design. A computer generated randomization sequence will assign schools to simple or complex treatment.

5.3 Implantation of Study Device

Silver diamine fluoride, along with all fluoride varnish materials are stored in a locked closet at NYU Dentistry. As indicated in 5.1, after drying the affected tooth surface, silver diamine fluoride is applied with an Advantage Arrest Applicators for approximately 30 seconds. The appropriate fluoride varnish is then applied to all teeth.

5.4 Subject Compliance Monitoring

N/A

5.5 Prior and Concomitant Therapy

Prior therapy is noted on the clinical examination. The only concomitant therapy provided in this study is fluoride varnish. All children are referred to a local dentist or community health center for follow on care. However, our experience is that only approximately 10% of parents follow through. Therefore, we see children once or twice per year. Using complex prevention, we achieve ~50% reduction in untreated caries, and almost complete elimination of toothaches.

5.6 Packaging

Silver diamine fluoride is available in an 8 oz. plastic bottle. One to 2 drops of liquid are dispensed into a dappen dish for delivery to the affected area using Advantage Arrest Applicators.

5.7 Blinding of Study

Neither the participants or clinicians are blinded. The analysts, however, are blinded.

5.8 Receiving, Storage, Dispensing and Return

5.8.1 Receipt of Study Device

Upon receipt of the of the silver diamine fluoride, an inventory will be performed and a device receipt log filled out and signed by the person accepting the shipment. The designated study staff will count and verify that the shipment contains all the items noted in the shipment inventory. Any damaged or unusable study devices in a given shipment will be documented in the study files. The investigator will notify the study sponsor of any damaged or unusable study devices that were supplied to the investigator's site.

5.8.2 Storage

Silver diamine fluoride will be stored with other dental supplies in a locked closet on the 8th floor at NY. Do not freeze.

5.8.3 Dispensing

A drop of silver diamine fluoride is dispensed from the 8 ml plastic bottle into a dappen dish prior to application for each patient. Advantage Arrest Applicators are used to apply the solution to pits, fissures, and frank caries for 30 seconds. Following application of the silver diamine fluoride varnish is applied.

5.8.4 Return

Typically, we completely use all of an 8 ml plastic bottle each day, as a bottle provides enough liquid to care for approximately 150 children. We dispose of the empty or partially empty bottle each day.

6 Study Procedures

6.1 Description

6.1.1 Informed Consent

All students enrolled in the 76 participating elementary schools, with paper or electronic informed consent signed by the parent/guardian, will receive caries prevention currently recommended by systematic reviews and national guidelines. Care will be provided independently of a family's ability to pay. Informed consent will be active for the entire time the child is in the school. Each year any newly enrolled students at the school will have the opportunity to join the program and be enrolled in the study if the parent/guardian signs the paper or electronic informed consent. If guardianship of the child changes during this time a new paper or electronic informed consent will need to be signed. The consent form is valid for 5 years or as long as the child is in NYC schools.

To facilitate accurate informed consent and student tracking we will request student rosters from the NY City Department of Education (NYC DOE). This will facilitate tracking students longitudinally. All forms will be translated into the major languages of NYC school children's parents. Those languages are Arabic, Bengali Bangla, Chinese, English, French, Haitian Creole, Korean, Russian, Spanish and, Urdu.

Blank IRB-approved paper informed consent will be distributed during Caried*Away* community engagement events and or mailed to students at the beginning of each school year in concert with all other school notifications. Parents can also choose to sign their child up for the program at their child's school. The current IRB approved blank informed consent will include a pre-populated personalized principal recruitment letter from the principal, the parent/guardian alternate location recruitment letter, the parent/guardian recruitment flyer, program information, a health history, the NYU Langone Health COVID-19 research participant information sheet and, the blank informed consent to be filled out for signature by the parent/guardian.

Completed parent/guardian signed and dated paper informed consent will be returned to the school and collected by the research team. This protocol aligns with other school vision, hearing, and vaccination programs. Care will be

provided to all children with informed consent. The information listed on the signed paper informed consent is loaded directly into the Apple password protected iPad to create the child's secure electronic dental record.

Electronic informed consent forms will be distributed in addition to the established protocol for the distribution of paper consent forms to participating sites (schools). The following protocol will be followed for eICF distribution:

Electronic informed consent can be distributed by a clinical team member using the Caried*Away* new enrollee email written in English or Spanish using the following hyperlinks:

- English language eICF: https://is.gd/CariedAwayNYC
- Spanish language eICF: https://is.gd/CariedAwayNYC_SP

Electronic informed consent can be distributed by the schools in English or Spanish using whichever existing method the school uses for mobile phone contact, in emails, or on the school's social media page in the following ways:

1) NYU Caried*Away* Website URL by selecting "registering your child"
   1. English
      - https://dental.nyu.edu/faculty/epidemiology-health-promotion/pcori-grant.html
      - Short URL: https://is.gd/CariedAwayNYC
   2. Spanish
      - https://dental.nyu.edu/faculty/epidemiology-health-promotion/pcori-beca.html
2) Hyperlinks in English and Spanish
   - English language eICF: https://is.gd/CariedAwayNYC
   - Spanish language eICF: https://is.gd/CariedAwayNYC_SP

The parent or legal guardian fills out the eICF and uses 'scratch' method (mouse, stylus, or finger) to sign and then returns the completed ICF via Redcap.

Only select study investigators will have access to the eICF data, which will be stored on REDCap. This includes the principal investigators, the clinical research coordinator, the study administrator, the clinical director, the IRB coordinator and NESS.

The study administrator will then cross-reference the OSIS number in the original .csv file which was provided to us at the beginning of each school year by the NYC Department of Education, and verify that the correct OSIS number was provided correctly by the consenter.

The study administrator then uploads all of the informed consent PDF's to NESS via the secure and password protected ShareFile link listed below:

- https://nedental.sharefile.com

NESS then uploads each student's eICF to their electronic dental record in the iPad.

6.1.2 *Scheduling*

For in school scheduling of preventive care our NYUCD HCP will work with the schools.

For scheduling at alternate community-based locations our clinical teams will work with the administrator at each community-based location.

Parents/guardians of children who have consented and need to be scheduled at a community-based location will be contacted via telephone by the NYUCD HCP to schedule an appointment. They will be provided with the name and address of where we will be providing alternate care and a list of dates and times for scheduling. If they are unavailable to schedule based on the dates provided, we will contact them when new dates and times become available.

6.1.3 *COVID-19 Pre-Screening and Screening Procedures*

The following procedures are performed

6.1.3.1 *NYUCD HCP Screening*

All NYUCD HCP are required to complete the COVID-19 NYU daily screener. Any NYUCD HCP who fails on the "screener pass" will automatically be referred to the NYU COVID prevention & response team and will not report to work.

6.1.3.2 *Screening In-school Students and School Staff*

We will follow the school's protocol for COVID-19 screening of students and school staff.

6.1.3.3 *Screening of Community-based Staff*

We will follow the community-based locations protocol for COVID-19 screening of their staff.

6.1.3.4 *Telephone Pre-screening of Children, Parent/Guardians at the Community-based Location*

1. 24 hours prior to the child's appointment the NYUCD HCP will telephone the parent/guardian and ask them the following questions:
   a. Have you or your child had close contact with anyone who has a confirmed or suspected COVID-19 diagnosis in the past 14 days
   b. Have you or your child had any flu-like symptoms like fever, coughing, shortness of breath or difficulty breathing in the past 14 days?
   c. Have you or your child had at least two of the following symptoms in the past 14 days: chills, repeated shaking with chills, muscle pain, headache, diarrhea, sore throat, new loss of taste or smell?

d. Have you or your child traveled within a state designated by New York State's Governor's office as a restricted state?

If the parent/guardian answers **yes** to any of the above questions, the child's appointment will be scheduled for a later date (10 or more days later)

6.1.4 Clinical Room Set-up

The following procedures will be performed

The clinical treatment room set up will vary based on room size and ability to provide care at a safe distance. If the clinical room is small, only 1 station will be set up to provide care to 1 child at a time. If the clinical room is large (eg: auditorium stage or large empty room), individual stations (up to 3) will be set up and spaced at a minimum of 6' apart.

The clinical work area is disinfected with an EPA registered intermediate level surface disinfectant before supplies needed for the day are removed from suitcases or backpack. Supplies needed for the day are placed on a barrier protected clean table at least 6' away from the dental chair. The table is covered with a vinyl tablecloth. The suitcase is then placed under the clean table for storage.

A barrier protected dirty table is set up at least 6' away from the barrier protected clean clinical supply table.
The dirty table is used for the disposal of used PPE, use disposable clinical supplies, and used non-disposable clinical supplies which are placed in a biohazard labeled sharps container and placed under the dirty table
Clinical Room Diagram: See illustration below:

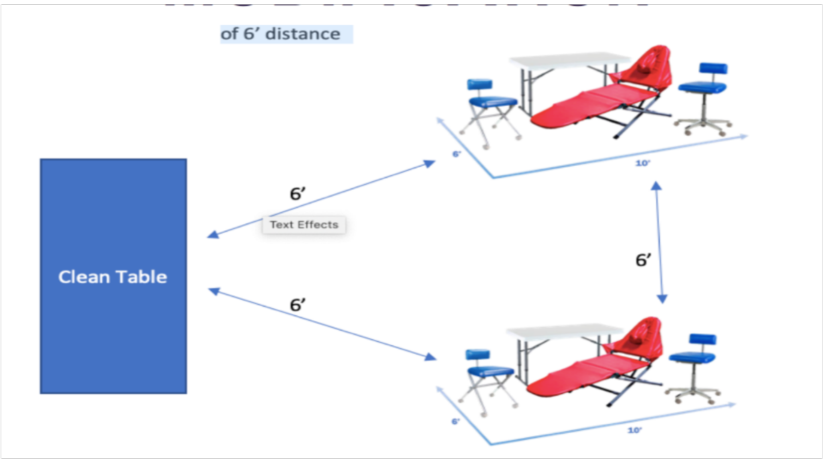

6.1.5 *Waiting Area*

The following procedures will be followed in-school or at a community-based location:

6.1.5.1 Waiting Area Preparation

The following procedures are performed:

1. On entry to the waiting area, a hand sanitation station is set up with:
   a. Alcohol-based hand sanitizer with 60-72% alcohol
   b. Disinfectant
   c. Contact free thermometer
2. Caried*Away* HCP will set up chairs for the waiting area ensuring a 6' distance.
3. Any toys, reading material, or any other objects that are difficult to clean will be removed.
4. A designated area will be assigned to perform the quality of life questionnaire (if applicable).

6.1.5.1.1 *Waiting Room Screening Procedures*

1. Upon arrival the dental assistant will confirm that the child and individual escorting them are wearing face masks or covering. If not, they will be supplied with a mask.
2. The dental assistant will ask the child and escort to apply hand sanitizer.
3. The dental assistant will take the temperature of the child and escort with a contact free thermometer. Note: If the child or escort has a temperature of 100.4 °F or greater they will be deferred to seek primary care evaluation and the appointment will be postponed.
4. If the child and escort has a temperature of less than 100.4 °F the dental assistant will escort the child to the treatment area where they will seat the child in the dental chair.

If appropriate, the person escorting the child will be seated in the waiting are with their mask on until the child has completed their treatment visit.

The waiting room seats will be spaced 6' apart and disinfected with an EPA registered intermediate level surface disinfectant between seating.

6.1.6 *Examination and Diagnosis*

Each program will use password-protected Apple iPads with electronic dental records pre-populated with demographic information of all students with informed consent. Student lists on the Apple iPads will be used to: collect students during the care delivery days; record clinical findings and care delivered; and generate take home forms (see below). On the scheduled care days, we will provide a gentle toothbrush cleaning, oral hygiene instruction, a toothbrush and fluoridated toothpaste. We will also carry out an examination and provide preventive care.

The examination will include a soft tissue inspection and an assessment of all primary and permanent teeth to determine the presence/ absence of decay (active or arrested), missing, filled, or sealed surfaces on all teeth.

6.1.7 *Prevention*

Both simple and complex care regimens provide both primary and secondary caries prevention. The goal of primary prevention is to prevent or delay the initial carious lesion using: 1) fluoride varnish for smooth surfaces (simple and complex prevention); and 2) either silver diamine fluoride (simple prevention) or sealants (complex prevention) for pits and fissures. The goal of secondary prevention is to arrest caries *in situ*, and prevent disease progression. This is accomplished with silver diamine fluoride (simple prevention) or interim therapeutic restorations (complex prevention).

6.1.8 *Take Home Messages and Follow-on Care*

We will provide two take-home messages, one for parents, and one for school nurses. The parents' message will identify the care provided to the child, note any additional care needed.

Parents of children without a dentist or children that require an urgent referral will be provided information on local dental providers in the following ways:

6.1.8.1 Children without a Dentist

Parents/guardians of children without a dentist can access the Caried*Away* Website Dentist finder map and link (https://dental.nyu.edu/faculty/epidemiology-health-promotion/pcori-grant.html). They are instructed to click on the map, type in their address and then click on the blue pins to access the name, address and phone number for that provider. All providers listed accept new patients, see children and, accept Medicaid and CHIP. Parents will also be provided with the NYU CariedAway- addition to Website Dentist Provider Map Instructions.

6.1.8.2 Children that require an urgent referral

Parents/guardians of children that require an urgent referral will receive the NYU Caried*Away* parent communication urgent referral email sent to them by the Caried*Away* clinical mangers. This email provides them with the CariedAway-addition to Website Dentist Provider Map Instructions and link (https://dental.nyu.edu/faculty/epidemiology-health-promotion/pcori-grant.html). The email also provides information for the NYU Department of Pediatric Dentistry.

The message for the nurses will be Excel spreadsheets that list the children seen, care provided and care needed, and that identify children who need immediate care. For the latter, we will follow each school's protocol.

6.2 Preventive Care

6.2.1 Simple prevention

Consists of a gentle toothbrush cleaning, oral hygiene instruction, an examination, delivery of silver diamine fluoride applied to all pits and fissures on bicuspids or molar teeth, and to all posterior, asymptomatic carious lesions. Fluoride varnish is then applied. Each child is given a small bag with a toothbrush, fluoride toothpaste, and a note indicating the results of the examination, the care provided, and a list of local dentists or community health centers for follow on care.

6.2.2 Complex prevention

Consists of a gentle toothbrush cleaning, oral hygiene instruction, an examination, delivery of glass ionomer sealants to all pits and fissures on bicuspids or molar teeth, and to all asymptomatic carious lesions. Each child is given a small bag with a toothbrush, fluoride toothpaste, and a note indicating the results of the examination, the care provided, and a list of local dentists or community health centers for follow on care.

6.3 Study Administration and Duration

Examination and care will be provided based in the school, at a community-based location, or NYUCD schedule and allow for a 30-minute eating free time following our care.
Care is delivered in a school room (e.g.: an empty room or class room, auditorium stage, nurse office, etc.), at a community-based location, or at NYUCD using mobile dental equipment and disposable supplies.

The clinical treatment room set up will vary based on room size and ability to provide care at a safe distance. If the clinical room is small, only 1 station will be set up to provide care to 1 child at a time. If the clinical room is large (eg: auditorium stage or large empty room), individual stations (up to 3) will be set up and spaced at a minimum of 6' apart.

The room is set up prior to the beginning of treatment.

All clinical personnel will wear personal protective equipment consistent with CDC, DOH, FDA and NYU Dentistry guidelines.

NYUCD licensed health professionals will be providing care and will also oversee all care that is provided by NYUCD senior dental hygiene students at all locations. All clinical personnel providing care will wear PPE based on current NYU Dentistry guidelines. PPE is listed below:

1. Disposable gowns
2. Disposable gloves
3. N95 respirator that have been fit tested for a specific respirator and shall be disposed of at the end of each clinical day
4. Level 3 surgical masks
5. Goggles
6. Face Shields
7. Bonnets
8. Booties

Gloves are changed between patients. All dental chairs, stools and touch surfaces are disinfected with an EPA registered intermediate level surface disinfectant at the beginning of each day, prior to and after treating patients, and at the end of the day. Goggles and face shields are also disinfected between patients.

Care is delivered only during school, community-based location, or NYUCD hours. Based on informed consents, children will be scheduled and seen in a school room, a community-based location room, or at NYUCD. Children are escorted to the waiting room in the school by the school's parent coordinator. Children are brought by the parent/guardian at a community-based setting or at NYUCD. After the child and parent/guardian at the community-based location passes the COVID-19 screening test, the child will be escorted to the clinical treatment room by the dental assistant at all locations.

Care will be provided by NYUCD supervising dentist, NYUCD registered dental hygienist, NYUCD senior dental hygiene student being overseen by NYUCD licensed health professionals or NYUCD registered nurse, with an assistant recording the examination and care delivery. After the child has completed treatment they will be required to put their mask back on and apply an alcohol-based hand sanitizer. The dental assistant will bring the child to the waiting room area. The child will be escorted back to the classroom by the school's parent coordinator or back home by the parent/guardian from the community-based, or NYUCD locations.

Simple prevention takes approximately 5 minutes and is aerosol free. It consists or a gentle tooth brush cleaning, oral hygiene instruction, an examination, delivery of silver diamine fluoride which takes approximately 1 minute to apply using Advantage Arrest Applicators to all pits and fissures on bicuspids or molar teeth, and to all posterior, asymptomatic carious lesions. Fluoride varnish is then applied which also takes 1 minute to apply.

Complex prevention takes approximately 20 minutes and is aerosol. It consists or a gentle toothbrush cleaning, oral hygiene instruction, an examination, delivery of glass ionomer sealants to all pits and fissures on bicuspids or molar teeth, and to all asymptomatic carious lesions. This takes about 5 minutes to provide care to ¼ of the mouth at a time. Fluoride varnish is then applied which takes approximately 1 minute.

6.4 Control/Comparison Group and Confounding Factors

Complex prevention is the "comparison" while simple prevention is the "intervention" group. Both groups receive primary and secondary prevention and we expect both protocols to be similarly effective in reducing untreated caries.

We will include relevant confounders a priori in our analysis, including: gender, previous (or concurrent) dental treatment (identified as new or existing treated dentition at examination), age at examination, race/ethnicity, and any school-level indicators. As these data have a multilevel structure, we will additionally assess caries incidence and prevalence using multilevel mixed effects Poisson and logistic modeling (ML-MEM). We will examine the effects of comprehensive prevention at multiple levels (tooth, child, grade, and school). In this analysis, we will be able to explore the variation in clinical outcomes across child and school levels. For all GEE and multilevel models, we will conduct analysis for outcomes (caries prevalence and incidence) measured by all teeth, all adult teeth only, and all deciduous teeth only.

6.5 Randomization and Blinding

6.5.1 Randomization

We will do a block randomization, generating sets of unique numbers to assign the experimental condition. Thus, for the first 30 schools we will generate 15 sets, each set containing a 1 or a 2, representing treatment assignment, in random order. We will repeat this in year 2 for the second set of 30 schools. In year 2, an additional 16 schools will be selected to receive simple prevention once per year.

6.5.2 Blinding

As a pragmatic trial, this is non-blinded for the participants. Neither schools, nor teachers, nor students, nor clinicians will be blinded. Clinicians will work in teams that only provide either simple or complex prevention. The analysts will be blinded as to the care provided to students in a particular school.

6.6 Compliance Assessment

This intervention trial depends on protocol compliance by clinicians, not students. We will train and calibrate clinicians during the summer before beginning each school year. To assess compliance, we will use qualitative and quantitative methods together with audit and feedback.

Qualitatively, to ensure that the electronic records reflect actual implementation, we will visit each team in the fall and spring of each year for a one-day site visit.

During the day, we will monitor adherence to protocol, particularly: student flow, caries diagnosis, and procedures delivered.

Quantitatively, we will use the elementary methods of statistical process control[27,28]. We will examine four domains: 1) informed consent rates; 2) caries prevalence (e.g.: diagnosis); 3) care provision; and 4) outcomes. We will monitor these domains using electronic records and then verify with in-person site visits. We will start with national averages and standard deviations for school-based informed consent rate (35%), untreated caries prevalence of (21.5%). All children (100%) with permanent bicuspids and molars should receive either silver diamine fluoride (simple prevention) or traditional sealants (complex prevention) and fluoride varnish. All children (100%) with caries on their posterior teeth should receive either silver diamine fluoride (simple prevention) or interim therapeutic restorations (complex prevention).

We will create histograms, Pareto charts, and control charts, at the school, grade, and clinician level to identify common cause and special cause variation. Control limits will be adjusted yearly based on prior year data. If a clinician, school or grade exhibits variation that is out of the control range we will use this audit to work with the clinical team and school to provide feedback. We will continue this process during the school year.

6.7 Prior and Concomitant Therapy

NA

6.8 Receiving, Storage, Transporting, Dispensing, Breakdown and Disposal of Clinical Supplies

6.8.1 Receiving

The products used for this trial are typical standard of care (SOC) dental commodities obtained from Henry Schien and Elevate Oral Care. They include toothbrushes, toothpaste, bibs, gauze, q-tips, floss, fluoride varnish, silver diamine fluoride (a topical varnish used to treat and prevent dental caries and relieve dentinal hypersensitivity, and glass ionomer cement (a dental restorative material used in dentistry for dental fillings) and all personal protective equipment (PPE). They are shipped to NYUCD and stored in a locked closet until used on the 8th floor with other clinical supplies. The same products will be safely transported to the participant schools or the local community-based location for study use.

6.8.2 Storage

All supplies when not in use are stored in a locked closet on the 8th floor at NYUCD.

6.8.3 Transporting

Supplies are transported to and from the schools or community-based location in locked roller suitcases or locked backpacks. Non disposable contaminated instruments are placed in a lockable puncture resistant plastic container which is labeled with a biohazard sticker. The outside of the container is wiped down with an EPA registered intermediate level surface disinfectant. Disposable sharp instruments are then placed in red biohazard bags, with a biohazard label affixed

to the outside of the bag. The bag is then transported in a combination locked Agnus Bag (suitcase) to NYU for disposal per NYUCD protocol.

6.8.4 Dispensing

All supplies are removed from locked roller suitcases or locked backpacks. Using clean gloves clinical supplies are placed on a clean barrier protected table at least 6' apart from the treatment station and barrier protected dirty table.

6.8.5 Breakdown

After completing treatment, all non-used clinical supplies from the clean table are placed back in the suitcase or backpack. The outside of the suitcase and backpack are disinfected with an EPA registered intermediate level surface disinfectant and then locked and transported back to NYUCD where they are placed in a locked closet on the 8th floor.

6.8.6 Disposal

After each individual procedure is performed disposable items are brought to the barrier protected dirty table which is at least 6' away from the barrier protected clean table. Disposable items are placed in the trash. All non-disposable sharps are placed in the sharps container labeled with a biohazard sticker which is located under the barrier protected dirty tale. Solid waste is disposed of into the receptacles lined with clear plastic bags. If any of the materials contain or may contain absorbed droplets of blood, they are placed in a red biohazard bag with a biohazard label affixed to the outside of the bag. The bag is then transported in a combination locked Agnus Bag (suitcase) to NYU for disposal per NYUCD protocol.

6.9 Study Procedures Table

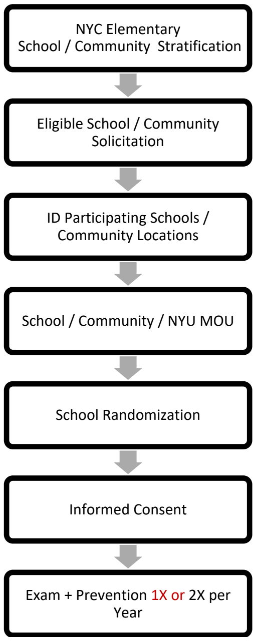

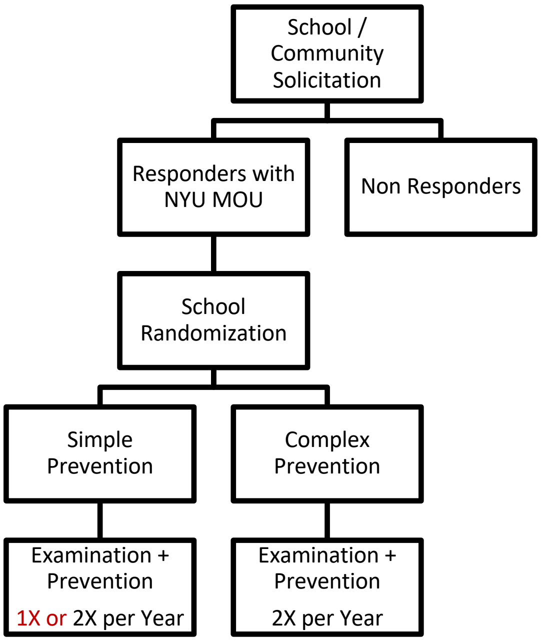

Following stratification and solicitation, execution of MOU with individual schools, community-based organizations, and randomization, care will be provided in a school, at a community-based location, or at NYUCD. In year 1 care will be offered in 30 schools, and in year 2 an additional 46 schools. In year 3 care will also be offered at a community-based location and at NYUCD.

7 Statistical Plan

7.1 Sample Size Determination

The study is powered to the primary outcomes of caries arrest and caries prevention between twice-yearly simple and complex prevention. We expect to enroll approximately 14,100 students across the initial 60 schools over the duration of the study (N=235 per cluster). The remaining 16 schools will contribute approximately 3760 participants. From our pilot studies of school-based caries prevention, baseline caries prevalence is approximately 40%. Our power estimates assume an equal proportion of success, π, of 20% caries arrest. With a given non-inferiority margin (δ) of 10%, a total sample size per group of 198 (N_{tot} = 396) is required for an alpha of 5% and a power of 80%[29] . However, as we use a cluster randomized design, this sample size is inflated to account for clustering (intraclass correlation coefficient = .10) by a design effect of 24.4 to a total required sample of 9,662.

For caries prevention, both interim therapeutic restorations and silver diamine fluoride have been shown to be 80% efficacious in individual clinical trials. Power for the repeated measures design was estimated using the method of Diggle et al (2002) for generalized estimating equations [30]. For power estimates, we control for the baseline prevalence of untreated decay. We assume a conservative average number of visits per child of 6, with a power of .80 and an alpha of 5%. We further assume a repeated measures correlation of 0.5 and a per-visit attrition rate of 20%. For a given minimally detectable effect size (standardized effect size difference) of .25, an attrition and clustering adjusted sample size of 12,874 is required. Thus, our study is powered for these conservative assumptions for caries prevention. Further, we note that in the presence of the nonlinear link function, ME-GLM is more powerful than GEE, thus our power estimates are conservative and the anticipated sample size is sufficient for analysis.

7.2 Statistical Methods

For caries arrest, we will first determine the per-patient proportion of carious lesions treated with simple versus complex prevention that stayed arrested throughout the length of the study. The percentage of arrested caries (at the child level) will be modeled using multilevel binomial regression. Our noninferiority margin, δ, is set at 10%. Our null hypothesis is that simple prevention is not inferior to the complex prevention by at least δ: $\pi_{simple} - \pi_{complex} \geq \delta$. Our alternative hypothesis is that $\pi_{simple} - \pi_{complex} < \delta$. We will use differences in effect sizes as estimated by confidence intervals to determine clinical non-inferiority of the two prevention methods. For the prevention of new caries, we will use generalized estimating equations (GEE) with a logit link (caries prevalence) and a negative binomial link (caries incidence), assuming an exchangeable correlation matrix, to evaluate longitudinal effects of comprehensive care untreated decay. We will identify the number of teeth at risk for each child during each follow-up interval and determine the number of those teeth in which new caries is observed at the examination that ends that interval. Primary teeth lost in each interval and new permanent teeth will not contribute to data for that interval. To explore non-linear trends in untreated decay between simple and complex prevention, we will use generalized additive models (GAMs) with non-parametric smoothers. Longitudinal effects of simple and complex prevention on academic outcomes, compared to untreated children, will be analyzed using propensity score-matching and multilevel modeling. First, we will estimate propensity scores for each participant at baseline, establishing the probability of treatment assignment conditional on observed covariates (e.g., prior academic performance). Propensity scores will be used to match treatment students to students not receiving treatment, considering multiple forms of matching

such as nearest neighbor and caliper. Treated students and matched comparators will then be analyzed using multilevel mixed effects linear regression (for academic achievement) and Poisson regression (for school absences).

Longitudinal effects of simple and complex prevention on dental-related service use and costs among Medicaid participants, compared to children in non-participating schools, will be analyzed using difference-in-differences analysis and propensity score matching. The propensity score weighting will be used to match children in participating schools to children in non-participating schools to account for any selection impacting whether a child is at a school participating in the Caried*Away* programs. The propensity score weighting will be used to account for student-level and school-level characteristics impacting whether the student is at a school participating in the Caried*Away* programs.

We will then adapt a published Markov model in combination with threshold analysis to compare the simple and complex prevention programs relative to non-participating schools in net costs and quality-adjusted life-years to determine the cost-effectiveness and budget impact of all three options (simple prevention, complex prevention, no prevention).

7.3 Subject Population for Analysis

For study outcomes, participants will be analyzed using intent to treat. All participants will be analyzed as randomized in the original randomization, regardless of whether or not they received an intervention.

For dental-related service use and costs, all New York City elementary school-age children enrolled in Medicaid will be analyzed using intent-to-treat.

7.4 Interim Analysis

There are no interim analyses in this study.

8 DATA AND SAFETY MONITORING PLAN (DSMP)

All protocol amendments, other than minor administrative changes will be submitted in a prospective manner to the NYU School of Medicine IRB for approval and PCORI for notification.

8.1 Informed Consent

Each participating school will obtain consent in accordance with their local circumstances.

The eICF forms were created and are stored using the secure REDCap data management system provided by the NYU Clinical and Translational Science Institute. The REDCap system is HIPAA compliant.

The study administrator uploads all of the informed consent PDF’s to NESS via the secure and password protected ShareFile link listed below:

- https://nedental.sharefile.com

NESS then uploads each student’s eICF to their electronic dental record in the iPad.

Only select study investigators will have access to the eICF data, which will be stored on REDCap. This includes the principal investigators, the clinical research coordinator, the study administrator, the clinical director, the IRB coordinator and NESS.

8.2 Risk

Risks associated with program participation include potential loss of privacy of the data. The strict security and Data Monitoring plan outlined in this proposal will help reduce this potential risk. Monitoring will be carried out by the PIs and Program Director.

8.3 Implementation

On a daily basis, performance sites will upload data to New England Survey Systems (NESS). On a weekly basis, NESS will upload data to the Biostatistics and Epidemiology Data Analytics Center (BEDAC) formerly the Boston University Data Coordinating Center (DCC). The BEDAC will maintain records for each performance site that includes patients seen and data. The BEDAC will provide weekly reports to the PIs, Program Director, and clinical research associate (CRA) for each performance site that includes the daily reports. These reports will be matched with the MOPs to ensure timely recruitment and data reporting. If results are out of expected range, the CRA will contact the clinical team to determine the cause and develop a plan to resolve the problem. Monthly, quarterly, and yearly reports will be generated by the BEDAC and provided to the PIs and Program Director. The initial reports will include recruitment data, while subsequent reports will include recruitment, retention, and databases for analysis.

8.4 Confidentiality

8.4.1 Protection of Subject Privacy

Subject Confidentiality is strictly held in trust by the investigators, study staff, and the sponsor(s) and their agents.

The study protocol, documentation, data, and all other information generated will be held in strict confidence. No information concerning the study or the data will be released to any unauthorized third party without prior written approval of the sponsor.

All patient information will be stored on password protected tablet (Apple iPad) computers. The iPads are transported by our clinical staff in a locked backpack to and from treatment locations. The iPads are locked each night in a closet on the 8th floor at NYUCD. Participant data is uploaded at the end of each day through a secure transmitting server at NESS. NESS verifies validity of data and erases data from the tablet (Apple iPad). The BEDAC and NESS have additional data security protocols in place for data fidelity and security. Finally, NESS provides de-identified data to the BEDAC and the BEDAC provides de-identified data to the analytic team. Data monitoring, quality assurance, and quality control will be performed by the study Principal Investigators, NESS, the BEDAC, and the NYU Institutional Review Board.

The study monitor or other authorized representatives of the sponsor may inspect all study documents and records required to be maintained by the investigator, including but not limited to clinical data for the study subjects. The PI site will permit access to such records.

The Health Evaluation and Analytics Lab at New York University's Wagner School of Public Service and New York University's School of Medicine will act as a third-party to carry out the the linkage between the oral health outcomes data collected through Caried*Away* and New York Medicaid claims, encounter, and eligibility data. The linkage will be carried out using the Medicaid billing IDs collected through the implementation of the Caried*Away* program and/or first

name, last name, date of birth, gender and residential zip code of the student. The Health Evaluation and Analytics Lab (HEAL) will then remove all "facial" identifiers (IDs, addresses, etc.) from the data provided for analysis to construct a HIPAA-defined "limited data set". We will exclude children from the analysis who did not consent to providing their oral health outcomes data and Medicaid claims data.

The Medicaid claims, encounter, and eligibility data (and any other data with personal identifiers) will be maintained on password-protected computers locked inside metal cabinets that would in turn be fastened to office infrastructure, inside locked offices in limited-access buildings. Additionally, the cage will be bolted down to the floor to prevent the physical removal of the hard drive and the data from the cage and from the office. These computers would not have any connection to the internet except briefly periodically to upgrade software. All data backups would be maintained on password protected encrypted external hard drives stored in locked cabinets in locked offices. These specifications are in accordance with the agreement between the Centers of Medicare and Medicaid Services, the NYU School of Medicine, and NYU Wagner for the release and analysis of the New York Medicaid claims data. The PD/PI will use only indirect patient identifiers to minimize the likelihood of identification. No attempts will be made to identify patients in the data, and in the case where subgroup analysis leads to small sample sizes at risk of identification, data will be aggregated or the subgroup enlarged to prevent identification from occurring.

8.4.2 Confidentiality During Adverse Event (AE) Reporting

AE reports and annual summaries will not include subject or group-identifiable material. Each report will only include the identification code.

8.5 Expected Risks

There are no expected risks associated with the clinical interventions proposed in this study. The only risk would be a data breach. The only possibility for a data breach would be the loss or theft of the tablet computers (Apple iPads) used for data collection. However, data is typically synced with NESS before the data collector leaves the school.

The iPads are transported by our clinical staff in a locked backpack to and from treatment locations. The iPads are locked each night in a closet on the 8th floor at NYUCD.

8.6 Adverse Event/ Unanticipated Problems

8.6.1 Definitions

8.6.1.1 Adverse Event (AE)

For the purposes of this study, we know of no adverse events related to the clinical interventions in the study. Adverse events would therefore include protocol violations and/or breaches of confidentiality. Breaches of confidentiality are unlikely given the above procedures in place. Protocol violations will be identified using audits and feedback of patient records to ensure protocols are being followed and any complaints from patients. Any instances of protocol violation or breach of confidentiality will be reported to the IRB and to PCORI.

8.6.1.2 Unanticipated Problems (UP)

Unanticipated problems will be recorded in the data collection system throughout the study.

8.6.1.3 Serious Adverse Event (SAE)

A serious adverse event (SAE) is one that meets one or more of the following criteria:

- Results in death
- Is life-threatening (places the subject at immediate risk of death from the event as it occurred)
- Results in inpatient hospitalization or prolongation of existing hospitalization
- Results in a persistent or significant disability or incapacity
- Results in a congenital anomaly or birth defect

An important medical event that may not result in death, be life threatening, or require hospitalization may be considered an SAE when, based upon appropriate medical judgment, the event may jeopardize the subject and may require medical or surgical intervention to prevent one of the outcomes listed in this definition.

8.7 Time Period and Frequency for Event Assessment and Follow-Up

The PI will record all reportable events with start dates occurring any time after informed consent is obtained until 7 (for non-serious AEs) or 30 days (for SAEs) after the last day of study participation. At each study visit, the investigator will inquire about the occurrence of AE/SAEs since the last visit. Events will be followed for outcome information until resolution or stabilization.

8.8 Characteristics of an Adverse Event

8.8.1 Relationship to Study Intervention

To assess relationship of an event to study intervention, the following guidelines are used:

1. Related (Possible, Probable, Definite)

    a. The event is known to occur with the study intervention.

    b. There is a temporal relationship between the intervention and event onset.

    c. The event abates when the intervention is discontinued.

    d. The event reappears upon a re-challenge with the intervention.

2. Not Related (Unlikely, Not Related)

    a. There is no temporal relationship between the intervention and event onset.

b. An alternate etiology has been established.

8.8.2 Expectedness of SAEs

The Study PIs will be responsible for determining whether an SAE is expected or unexpected. An adverse event will be considered unexpected if the nature, severity, or frequency of the event is not consistent with the risk information previously described for the intervention. No SAEs are expected for clinical interventions in this study.

8.8.3 Severity of Event

The following scale will be used to grade adverse events:

1. Mild: no intervention required; no impact on activities of daily living (ADL)
2. Moderate: minimal, local, or non-invasive intervention indicated; moderate impact on ADL
3. Severe: significant symptoms requiring invasive intervention; subject seeks medical attention, needs major assistance with ADL

8.9 Reporting Procedures

8.9.1 Reporting for Multi-Center Trials

Not applicable.

8.9.2 Unanticipated Problem Reporting

Incidents or events that meet the Office of Human Research Protections (OHRP) criteria for unanticipated problems will result in completing an unanticipated problem report form. The following information will be reported when reporting an adverse event, or any other incident, experience, or outcome as an unanticipated problem to the IRB:

- Appropriate identifying information for the research protocol (title, investigator’s name, and the IRB project number);
- A detailed description of the adverse event, incident, experience, or outcome;
- An explanation of the basis for determining that the adverse event, incident, experience, or outcome represents an unanticipated problem;
- A description of any changes to the protocol or other corrective actions that have been taken or are proposed in response to the unanticipated problem.

To satisfy the requirement for prompt reporting, unanticipated problems will be reported using the following timeline:

- Unanticipated problems that are serious adverse events will be reported to the IRB and PCORI within 7 days of the investigator becoming aware of the event.

Any other unanticipated problem will be reported to the IRB and PCORI within 14 days of the investigator becoming aware of the problem.

8.9.3 Adverse Event Reporting of Non-IND Studies

SAEs that are unanticipated, serious, and possibly related to the study intervention will be reported to the IRB and PCORI in accordance with requirements.

- Unexpected fatal or life-threatening AEs related to the intervention will be reported to the PCORI Project Manager within 3 days of the investigator becoming aware of the event. Other serious and unexpected AEs related to the intervention will be reported within 7 days.
- Anticipated or unrelated SAEs will be handled in a less urgent manner but will be reported to the IRB in accordance with their requirements and will be reported to PCORI on an annual basis.
- All other AEs documented during the course of the trial will be reported to PCORI on an annual basis by way of inclusion in the annual report and in the annual AE/DSMP summary which will be provided to PCORI.

We note that no clinical adverse events are expected.

8.9.4 Adverse Event Reporting of Investigation New Drug (IND) Studies

Not applicable.

8.9.5 Events of Special Interest (if applicable)

Not applicable.

8.9.6 Reporting of Pregnancy

Not applicable.

8.10 Halting Rules

Given the 25-year history of the study PIs, we know of no safety issues that would prompt suspension of enrollment or the interventions. However, if we encounter serious, unexpected, and related AEs, the PIs, PCORI, and the IRB would confer to determine whether findings might trigger a safety review based on the number of overall SAEs, the occurrence frequency of the type of SAE, severe AEs/reactions, or increased frequency of events.

8.11 Quality Control and Quality Assurance

Each year of the study, student rosters in participating schools will be requested from the NYC Department of Education, which will include student unique identification numbers, names, addresses, and socio-demographic information.

At the end of each clinic day, the clinical lead in each treatment location will synchronize Apple iPads, used to collect data, with NESS to securely upload examination and clinical data to electronic dental records. Records will then be checked to verify successful transmission, and then erased from each Apple iPad. Transmitted data will include unique identifiers of study participants, demographic information, and examination and care data.

Congruence of unique identifier with demographics (for returning students), as well as in/out of range includes: date of birth with grade, presence/absence and number and type of teeth, increasing occurrence of permanent teeth and decreasing occurrence of primary teeth, and

logic errors (e.g.: change from decay to sound). Records with apparent errors will be set aside for hand check. If errors cannot be rectified, a child's data for a given visit will be excluded from the analysis.

If metrics are beyond established control limits, the CRA will contact the performance site. We will determine if there is special or to determine the cause and see if and how we can assist the performance site.

As indicated in the Research Plan, reports will be generated by the BEDAC and provided to the Co-PIs and Patient and Stakeholder Partners.

8.12 Subject Accrual and Compliance

NESS maintains records for each program, school, grade, and child. NESS provides weekly reports to the CRA (who will share this with the Co-PIs) to assess accrual. These reports will be matched with the manual of procedures for the performance sites to insure timely recruitment and data reporting.

As indicated in the Research Plan, reports will be generated by the BEDAC and provided to the Co-PIs and Patient and Stakeholder Partners.

8.12.1 Measurement and Reporting of Subject Accrual

NESS maintains records for each program, school, grade, and child. NESS provides weekly reports to the CRA (who will share this with the Co-PIs) to assess accrual. These reports will be matched with the manual of procedures for the performance sites to insure timely recruitment and data reporting.

As indicated in the Research Plan, reports will be generated by the BEDAC and provided to the Co-PIs and Patient and Stakeholder Partners.

8.12.2 Measurement and Reporting of Participant Adherence to Treatment Protocol

Not applicable.

8.13 Stopping Rules

This study will be stopped prior to its completion if: (1) the intervention is associated with adverse effects that call into question the safety of the intervention; (2) difficulty in study recruitment or retention will significantly impact the ability to evaluate the study endpoints; (3) any new information becomes available during the trial that necessitates stopping the trial; or (4) other situations occur that might warrant stopping the trial.

8.14 Safety Review Plan

Study progress and safety will be reviewed monthly. Progress reports, including patient recruitment, retention/attrition, and AEs will be provided to the community stakeholder groups as listed in the study protocol. An Annual Report will be compiled and will include a list and summary of AEs. In addition, the Annual Report will address (1) whether AE rates are consistent with pre-study assumptions; (2) reason for dropouts from the study; (3) whether all participants met entry criteria; (4) whether continuation of the study is justified on the basis that additional data are needed to accomplish the stated aims of the study; and (5) conditions whereby the study might be terminated prematurely. The Annual Report will be sent to IRB and

PCORI. The IRB and other applicable recipients will review progress of this study on an annual basis

Study Report tables will be generated only from aggregate (not by group assignment) baseline and aggregate safety data for the study population.

8.15 Submission of On-Site Monitoring/Audit and Inspection Reports

Any expectation of violations of protocols will result in an audit of data collection and study assessors. The IRB will receive copies of all study monitoring/audit reports that are submitted quarterly as listed in Table A.

Table A

Data type	Frequency of review	Reviewer
Subject accrual (including compliance with protocol enrollment criteria)	Monthly	PI, Internal QA Reviewer (BEDAC)
	Quarterly	Community Stakeholder Groups
Status of all enrolled subjects, as of date of reporting	Monthly	PI, Internal QA Reviewer (BEDAC)
	Quarterly	Community Stakeholder Groups
Data entry quality control checks on 100% of charts	Weekly	QA Reviewer (BEDAC)
Adherence data regarding study visits and intervention	Weekly	PI, Internal QA Reviewer
	Quarterly	Community Stakeholder Groups
Protocol violations	Weekly	PI, Internal QA Reviewer
	Quarterly	Community Stakeholder Groups
	Annually	PCORI, IRB
SAEs (unexpected and related)	Per occurrence	PI<br>PCORI
SAEs (expected or unrelated)	Per Occurrence	PI, Internal
	Annually	Community Stakeholder Groups<br>PCORI
Unanticipated Problems	Monthly	PI, Internal QA Reviewer
	Per Policy	IRB

8.16 Data Handling and Record Keeping

The investigators are responsible for ensuring the accuracy, completeness, legibility, and timeliness of the data reported. All source documents should be completed in a neat, legible manner to ensure accurate interpretation of data.

8.17 Data Management Responsibilities

Data collection and accurate documentation are the responsibility of the study staff and NESS, under the supervision of the investigators. All source documents and reports must be reviewed

by the study team and data entry staff, who will ensure that they are accurate and complete. Unanticipated problems and adverse events must be reviewed by the investigator or designee.

8.18 Database Protection

This study will incorporate a Biostatistics and Epidemiology Data Analytics Center located at Boston University for data storage and security. Access to the database will be secured with password protection. The informatics manager will receive only coded information that is entered into the database under those identification numbers. Electronic communication with outside collaborators will involve only unidentifiable information. The database incorporates an electronic audit trail to show change(s) to data after original entry including the date/time and user making the change.

8.19 Source Documents/Protections

Source data is all information, original records of findings, observations, or other activities in a clinical trial necessary for the reconstruction and evaluation of the trial. Source data are contained in source documents. For this trial the source documents include: informed consent, health history, examination and clinical care records. These are all collected and entered electronically on Apple iPads and maintained on a secure data base at NESS.

The eICF forms were created and are stored using the secure REDCap data management system provided by the NYU Clinical and Translational Science Institute. The REDCap system is HIPAA compliant.

The tablet computers (Apple iPads) used in this study are password protected for each user (with individual usernames and passwords). Data is transmitted over a secure private network. Data is transmitted to investigators using a secure 128-bit encryption.

When a staff member employed at NYUCD who has access to the Apple iPads is no longer employed by NYUCD, the CRA notifies NESS and that user's username and password is removed.

8.20 Case Report Forms

All study forms are electronic and reside on Apple iPads. Forms require completion prior to moving to next steps. Out of range entries are flagged immediately for correction, if necessary, and re-entry.

8.21 Records Retention and Storage

All records are securely stored electronically on the NESS servers. They will be stored for the term of the study.

8.22 Study/Regulatory Binder

We will use the MedPoint electronic study/regulatory binder: **https://esafe.medpt.com**

8.23 Schedule and Content of Reports

Enrollment reports will be reviewed biannually, as study enrollment will be conducted in the fall semester of each school year. Review will thus occur in the spring of the same academic year. Enrollment reports and other study reports will be presented to study stakeholder groups on a quarterly basis, as will reports of study conduct. All data will be masked to study investigators as

data are de-identified at the BEDAC prior to being sent to investigators for analysis. The study PIs and biostatisticians do not have access to the identification code key. Finally, participating schools and students will be assigned IDs prior to data collection.

8.24 Informed Consent

This study requires an alteration of informed consent. The Caried*Away* study team will not meet directly with the parent/guardian to discuss and obtain informed consent for their child to participate in this study. The consent process itself does not require a study team member to sign off on the informed consent. Instead, school administrators send and provide study information and informed consent for the parent/guardian to review and sign themselves. If the parent/guardian has any questions regarding the study or the informed consent process, they can contact the school administrator or the principal investigator directly.

Principal personalized pre-populated recruitment letters, the parent/guardian alternate location recruitment letter, the NYU Langone Health COVID-19 research participation information sheet and blank Informed consent forms are IRB-approved. The parent or guardian is required to read and review the documents or have the documents read to him or her. The parent or guardian will also have the opportunity to call the school administrator or the principal investigator if they have questions regarding the study. The investigator or designee will explain the research study and billing procedures to the school principal and designated staff at the initial recruitment meeting which may be conducted in person or via Zoom. Upon receiving approval from NYSDOH to provide services in schools, NYUCD HCP will meet again with the school principal and designated staff either in person or via Zoom to go over the program and discuss the next steps.

All parents/guardians of new or previously consented students will be contacted via telephone by one of our clinical team members prior to scheduling any appointments. Parents/guardians of previously consented students will be reminded that they signed a 5 year informed consent. The clinical team member will use the research participant telephone recruitment script for COVID-19 updates. They will ask them a few questions to assess whether it makes sense, from a safety perspective due to the COVID-19 pandemic if they wish to have their child to participate or continue to participate in this research study. After a clinical team member speaks with the parent/guardian of a new participant, the clinical team member will send the parent/guardian the Caried*Away* new enrollee email written in English or Spanish. The email will also contain a link to the REDCap eICF and provide contact information if the parent/guardian has further questions.

NYUCD Caried*Away* staff will also conduct the following community engagement events via Zoom, if possible. Actual activity will be guided by the signed MOU between Caried*Away* and the school or community organization. This may be modified on an ad hoc basis depending on the impact of, and reaction to, COVID-19 by a school or community location.

a. One professional development (PD) meeting at least per semester: NYUCD Caried*Away* staff will set up a Zoom meeting with each schools PD to educate teachers and additional staff about the program and billing procedures. NYUCD Caried*Away* staff will go over the personalized pre-populated principal recruitment letter, the parent/guardian alternate location recruitment letter which explains billing procedures and informed consent form in detail, discuss the process for scheduling students at alternate community-based locations and instruct all staff on how to properly fill out the informed consent form, and answer any questions they may have. Teachers will receive personalized pre-populated principal recruitment letters, the parent/guardian alternate location recruitment

letter, the NYU Langone Health COVID-19 research participation information sheet and blank informed consent forms to distribute to all of their students.

b. In-Class or Zoom Oral Health Education (OHE) Presentations at least once per semester: NYUCD Caried*Away* staff will provide in-class or Zoom OHE to each class at each school once per semester. This allows NYUCD Caried*Away* staff to engage with all teachers/staff as well as each student. Additional personalized pre-populated principal recruitment letters, the parent/guardian alternate location recruitment letter, the NYU Langone Health COVID-19 research participation information sheet and blank informed consent forms may be provided at this time.

c. Informational tabling at least once per semester: NYUCD Caried*Away* staff will provide parent/guardian informational tabling during student arrival and students dismissal time. This allows parents to meet NYUCD Caried*Away* staff, learn about the program, review and receive the personalized pre-populated principal recruitment letter, explain billing procedures, receive the informed consent form, and ask any questions they may have.

d. Completed signed and dated informed consent forms are returned to the designated school staff to be kept and checked for completeness. NYUCD Caried*Away* staff will collect these consents 2 weeks prior to a scheduled treatment week and ensure an additional check for completeness.

The parent or guardian will sign the completed paper or electronic informed consent document prior to any study-related assessments or procedures. Consent may be withdrawn at any time throughout the course of the study.

A separate picture of page 1 and page 2 of the completed, signed and dated paper informed consent is taken with the iPad camera and each image is saved and stored in the electronic dental record for each child in the manage subject page within the child's electronic dental record on the Apple iPad.

8.25 Reporting Changes in Study Status

During the funding of this study, any action by the IRB or one of the study investigators that results in a temporary or permanent suspension of the study will be reported to the PCORI Program Official within 3 business days of notification.

9 Ethical Considerations

This study will be conducted in compliance with the protocol approved by the IRB, GCP guidelines, and applicable NYUMC and federal regulatory requirements. No deviation from the protocol will be implemented without the prior review and approval of the IRB, except where it may be necessary to eliminate an immediate risk to a participant. In such case, the deviation will be reported to the IRB according to its policies and procedures.

This protocol and any amendments will be submitted to an IRB in agreement with local legal prescriptions, for formal approval of the study conduct. The decision of the IRB concerning the conduct of the study will be made in writing to the investigator.

This study involves children. All parents/legal guardian will be provided an assent form describing the study and providing sufficient information for them to make an informed decision about their participation. See Attachment ***[NYC DOE* CariedAway *Parental ICF]*** for a copy of the Participant Informed Consent Form. This consent form will be submitted with the protocol for review and approval by the IRB for the study. The formal consent of a participant, using the IRB-approved paper or electronic consent form, must be obtained before that participant undergoes any study procedure. The parent or a legally acceptable guardian or surrogate must sign the paper or electronic consent form.

10 Study Finances

10.1 Funding Source

This study is financed through a grant from the Patient Centered Outcomes Research Institute

10.2 Conflict of Interest

None.

10.3 Participant Stipends or Payments

None.

11 Publication Plan

Neither the complete nor any part of the results of the study carried out under this protocol will be published or passed on to any third party without the consent of the PI or primary responsible party. Any investigators involved with this study will be obligated to provide the PI or primary responsible party with complete test results and all data derived from the study.

12 Attachments

1. New York University / New York City MOU
2. Current New York City IRB approved Informed Consent

13 References

1. Dye B, Li X, Thornton-Evans G. Oral health disparities as determined by selected Healthy People 2020 Oral Health objectives for the United States, 2009-2010. *NCHS Data Brief.* 2012;104:1-8.

2. Pine CM, Harris RV, Burnside G, Merrett MC. An investigation of the relationship between untreated decayed teeth and dental sepsis in 5-year-old children. *British dental journal.* 2006;200(1):45-47; discussion 29.
3. Otto M. For want of a dentist. *The Washington Post.* February 28, 2007, 2007.
4. Farber, J., *Oral Health and the Commonwealth's Most Vulnerable Children: A State of Decay*. 2004: Boston, MA.
5. Blumenshine, S.L., et al., *Children's school performance: impact of general and oral health.* J Public Health Dent, 2008. **68**(2): p. 82-7.
6. Seirawan, H., S. Faust, and R. Mulligan, *The impact of oral health on the academic performance of disadvantaged children.* Am J Public Health, 2012. **102**(9): p. 1729-34.
7. Detty, A.M. and R. Oza-Frank, *Oral health status and academic performance among Ohio third-graders, 2009-2010.* J Public Health Dent, 2014.
8. Kelton, *31% of U.S. parents say kids missed school due to dental problems*. 2015, Delta Dental Plans Association: PR Newswire.
9. Niederman R. Bringing Care to People Rather Than People to Care. *Am J Public Health.* 2015:e1.
10. Niederman R, Huang SS, Trescher A-L, Listl S. Getting the incentives right: Improving oral health equity with universal school-based caries prevention. *American Journal of Public Health.* 2017 (in press).
11. Wong, M.C.M., et al., *Cochrane reviews on the benefits/risks of fluoride toothpastes.* Journal of dental research, 2011. **90**(5): p. 573-9.
12. Dos Santos, A.P., P. Nadanovsky, and B.H. de Oliveira, *A systematic review and meta-analysis of the effects of fluoride toothpastes on the prevention of dental caries in the primary dentition of preschool children.* Community Dent Oral Epidemiol, 2012.
13. Marinho, V.C.C., et al., *Fluoride varnishes for preventing dental caries in children and adolescents.* The Cochrane database of systematic reviews, 2013. **7**: p. CD002279.
14. Mickenautsch, S. and V. Yengopal, *Caries-preventive effect of glass ionomer and resin-based fissure sealants on permanent teeth: An update of systematic review evidence.* BMC research notes, 2011. **4**: p. 22.
15. Yengopal, V., et al., *Caries-preventive effect of glass ionomer and resin-based fissure sealants on permanent teeth: a meta analysis.* Journal of oral science, 2009. **51**(3): p. 373-82.
16. Ahovuo-Saloranta, A., et al., *Sealants for preventing dental decay in the permanent teeth.* The Cochrane database of systematic reviews, 2013. **3**: p. CD001830.
17. Ricketts, D., et al., *Operative caries management in adults and children.* The Cochrane database of systematic reviews, 2013. **3**: p. CD003808.
18. de Amorim, R.G., S.C. Leal, and J.E. Frencken, *Survival of atraumatic restorative treatment (ART) sealants and restorations: a meta-analysis.* Clinical oral investigations, 2012. **16**(2): p. 429-41.
19. Schwendicke, F., C.E. Dörfer, and S. Paris, *Incomplete caries removal: a systematic review and meta-analysis.* Journal of dental research, 2013. **92**(4): p. 306-14.
20. Rosenblatt, A., T.C.M. Stamford, and R. Niederman, *Silver diamine fluoride: a caries "silver-fluoride bullet".* Journal of dental research, 2009. **88**(2): p. 116-25.
21. Zhi, Q.H., E.C.M. Lo, and H.C. Lin, *Randomized clinical trial on effectiveness of silver diamine fluoride and glass ionomer in arresting dentine caries in preschool children.* Journal of dentistry, 2012. **40**(11): p. 962-7.
22. Liu, B.Y., et al., *Randomized trial on fluorides and sealants for fissure caries prevention.* Journal of dental research, 2012. **91**(8): p. 753-8.
23. Beltran ED, Malvitz DM, Eklund SA. Validity of two methods for assessing oral health status of populations. *J Public Health Dent.* 1997;57(4):206-214.
24. White JA, Beltran ED, Malvitz DM, Perlman SP. Oral health status of special athletes in the San Francisco Bay Area. *Journal of the California Dental Association.* 1998;26(5):347-354.

25. Petersen PE, Baez RJ. *Oral Health Surveys: Basic Methods.* Geneva: World Health Organization; 2013.
26. Dye BA, Barker LK, Li X, Lewis BG, Beltran-Aguilar ED. Overview and quality assurance for the oral health component of the National Health and Nutrition Examination Survey (NHANES), 2005-08. *J Public Health Dent.* 2011;71(1):54-61.
27. Benneyan JC, Lloyd RC, Plsek PE. Statistical process control as a tool for research and healthcare improvement. *Qual Saf Health Care.* 2003;12(6):458-464.
28. Provost LP, Murray SK. *The Health Care Data Guide. Learning from data for improvement.* San Francisco: Jossey-Bass; 2011.
29. Chow S, Shao J, Wang H. *Sample size calculations in clinical research* Boca Raton, Florida: Taylor & Francis; 2008.
30. Diggle PJ, Heagerty P, Kung-Yee L, Zeger SL. *Analysis of Longitudinal Data.* Oxford U.K.: Oxford University Press; 2002.
