# Supplementary material for: Effect of Silver Diamine Fluoride on Caries Arrest and Prevention: The CariedAway School-Based Randomized Clinical Trial
Source: JAMA Netw Open. 2023 Feb 9;6(2):e2255458. doi: 10.1001/jamanetworkopen.2022.55458 (PMC9912124; doi:10.1001/jamanetworkopen.2022.55458)
Supplement: Supplement 2. — Data Sharing Statement [file jamanetwopen-e2255458-s002.pdf]

Data Sharing Statement

Ruff. Effect of Silver Diamine Fluoride on Caries Arrest and Prevention. *JAMA Netw Open*. Published February 09, 2023. doi:10.1001/jamanetworkopen.2022.55458

Data
Data available: Yes
Data types: Data dictionary
How to access data: Data dictionaries will be available to interested researchers upon request to the authors (ryan.ruff@nyu.edu)
When available: beginning date: 09-01-2023

Supporting Documents
Document types: Informed consent form
How to access documents: Informed consent forms will be available to interested researchers upon request to the authors (ryan.ruff@nyu.edu)
When available: beginning date: 09-01-2023

Additional Information
Who can access the data: Interested researchers upon request to the authors (ryan.ruff@nyu.edu)
Types of analyses: For any purpose
Mechanisms of data availability: After approval of a proposal and a signed data access agreement.
